# Smart cellulose‐based room temperature phosphorescent materials: From mechanisms to construction strategies and applications

**DOI:** 10.1002/smo2.70059

**Published:** 2026-05-18

**Authors:** Shasha Wu, Yifan Zhou, Wanjin Wu, Yan Tang, Mengfan Jing, Jianyu Zhang, Haoke Zhang, Zheng Zhao, Junwei Wang, Laurent Maron, Lijie Liu

**Affiliations:** ^1^ State Key Laboratory of Structural Analysis, Optimization and CAE Software for Industrial Equipment National Engineering Research Center for Advanced Polymer Processing Technology Zhengzhou University Zhengzhou Henan China; ^2^ College of Science Henan Agricultural University Zhengzhou Henan China; ^3^ Department of Polymer Science and Engineering Key Laboratory of Macromolecular Synthesis and Functionalization Zhejiang University Hangzhou China; ^4^ School of Science and Engineering Shenzhen Institute of Aggregate Science and Technology The Chinese University of Hong Kong Shenzhen Shenzhen Guangdong China; ^5^ College of Energy Materials and Chemistry Inner Mongolia University Hohhot China; ^6^ Université de Toulouse INSA Toulouse CNRS LPCNO Toulouse France

**Keywords:** cellulose, cluster‐triggered emission, room temperature phosphorescence

## Abstract

Room temperature phosphorescent (RTP) molecules, owing to their unique afterglow characteristics, can translate microscopic electronic transitions into macroscopic smart responses, and have gradually emerged as a research hotspot in the field of smart molecules. Benefiting from the renewability, biocompatibility, and structural tunability of cellulose, these materials provide an ideal and sustainable platform for constructing RTP systems. Significant progress has been made in this field, leading to the emergence of numerous cellulose‐based RTP material systems with novel structures and excellent performance. This review summarizes recent advances in RTP systems cellulose derivatives. First, it elucidates the underlying mechanisms of cluster‐triggered emission, followed by a detailed discussion of four key construction strategies: regulation of aggregation structures, reconstruction of clustered emission centers, hydroxyl functionalization, and host‐guest doping. In addition, innovative applications in information security and environmental monitoring are highlighted. Finally, current challenges are discussed, and perspectives on the rational design of future biomass‐based RTP materials are provided.

## Graphical abstract

This review elucidates the photophysical mechanisms of cellulose‐based room temperature phosphorescent materials and systematically outlines their construction strategies, such as cluster emission center engineering. This article demonstrates their applications in fields like information encryption and bioimaging, and looks ahead to the core challenges in future development.

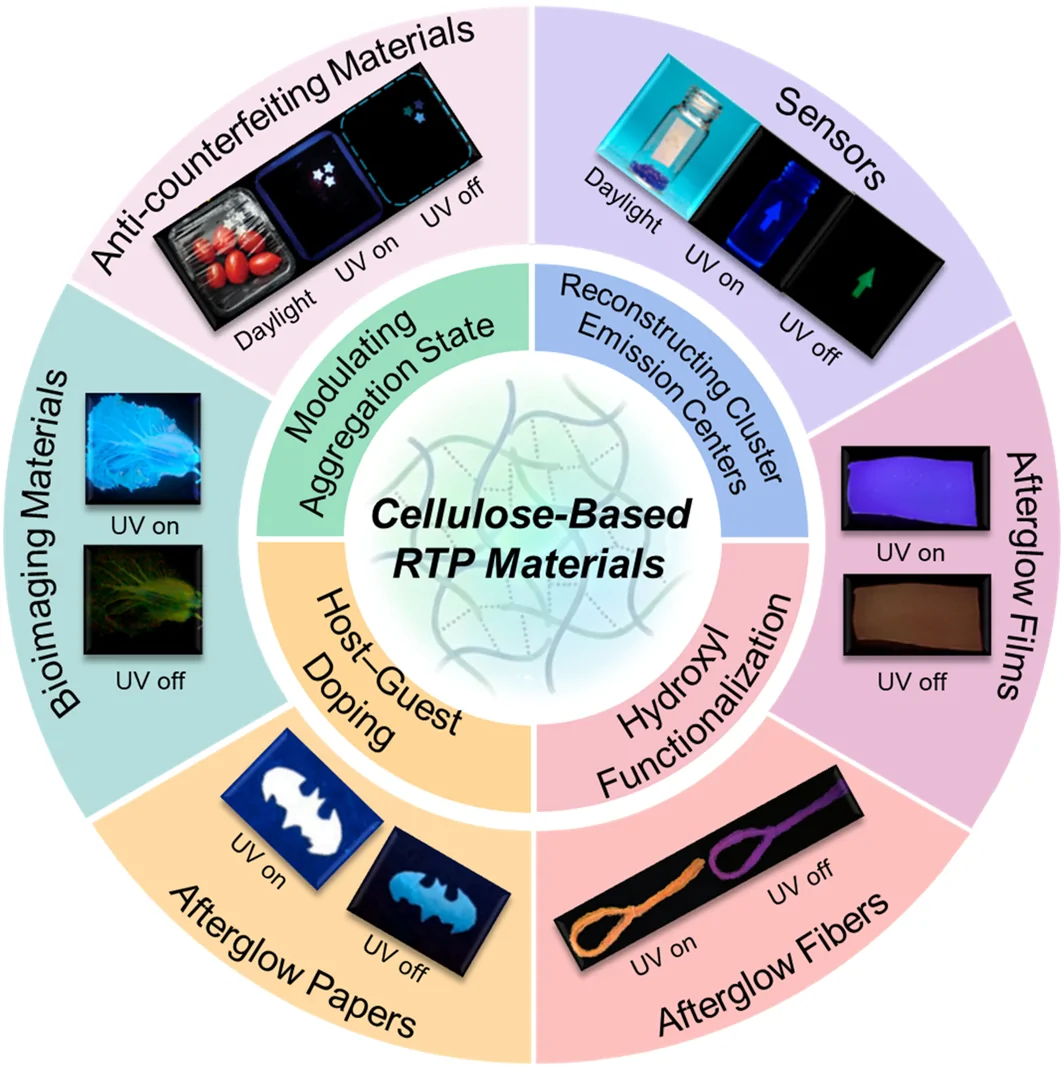

## 1 INTRODUCTION

Smart materials can exhibit predictable and reversible responses to external stimuli (such as light, heat, electricity, magnetism, etc.), enabling dynamic regulation and monitoring of their functions.[^1^, ^2^, ^3^] They are gradually permeating cutting‐edge fields such as aerospace, biomedicine, flexible electronics, and energy storage.[^4^, ^5^, ^6^, ^7^] Smart luminescent materials, owing to their unique optical responsiveness and information visualization capabilities, have become one of the most prominent research directions in recent years.[^8^, ^9^, ^10^, ^11^, ^12^] These materials can not only “sense” environmental changes but also “express” information through alterations in emission color, intensity, or lifetime, opening up new pathways for sensing, anti‐counterfeiting, bioimaging, and smart displays.[^13^, ^14^, ^15^] Among them, phosphorescent materials stand out due to their long afterglow characteristics.^[16]^ Phosphorescence originates from the T_1_ → S_0_ transition, with lifetimes ranging from microseconds to seconds, or even longer.[^17^, ^18^, ^19^, ^20^] This property endows phosphorescent materials with irreplaceable advantages in applications such as time‐resolved imaging, afterglow displays, and anti‐counterfeiting labels.[^21^, ^22^, ^23^, ^24^] Traditional phosphorescent materials predominantly rely on precious metal complexes (such as iridium, platinum, ruthenium). Although they exhibit excellent performance, their high cost, resource scarcity, and poor biocompatibility limit their large‐scale application.[^25^, ^26^, ^27^] To overcome these bottlenecks, organic phosphorescent materials have emerged with advantages such as strong structural designability, low cost, and environmental friendliness.[^28^, ^29^, ^30^, ^31^] However, the relatively small spin‐orbit coupling (SOC) in organic molecular systems makes it difficult to efficiently generate and radiate triplet excitons, posing a challenge for achieving room‐temperature.[^32^, ^33^, ^34^]

Currently, organic Room temperature phosphorescent (RTP) materials are primarily categorized into two main classes: small‐molecule systems[^35^, ^36^, ^37^] and polymeric systems.^[38]^ Small RTP materials typically rely on crystalline or highly ordered solid‐state structures to effectively restrict intramolecular motions, thereby suppressing non‐radiative decay. In contrast, polymeric RTP materials demonstrate greater potential for practical applications owing to their excellent film‐forming ability, good solubility and processability, and scalability. Based on the structural features, polymeric RTP systems can be further divided into two subcategories: conjugated and nonconjugated types. Among them, nonconjugated polymeric RTP materials, despite their relatively recent emergence and incomplete mechanistic understanding, have gradually become a research hotspot in recent years due to their advantages such as low cost, excellent environmental sustainability, and good biocompatibility. Their phosphorescence emission typically originates from a cluster‐triggered emission (CTE) mechanism,[^39^, ^40^] wherein nonconventional chromophores containing π or lone pairs electrons undergo effective conjugation extension and conformational rigidification through electron sharing and electron cloud overlap in the aggregated state, thereby significantly enhancing the phosphorescence radiative process.[^5^, ^41^]

In nonconjugated polymer systems, the cellulose molecular backbone, enriched with uniformly distributed oxygen atoms (hydroxyl groups and ether linkages), endows cellulose with high crystallinity and excellent chemical modifiability.[^46^, ^47^] These features are particularly advantageous for simultaneously fulfilling the requirements of an electron‐rich environment and rigid molecular confinement essential for the construction of RTP.^[48]^ Cellulose‐based RTP primarily originates from clustered structures formed by hydroxyl groups and oxygen atoms acting as nonconventional chromophores in the aggregated state, where phosphorescence is enabled through the CTE mechanism.^[39]^ After years of development, cellulose‐based RTP materials have evolved into an important branch of organic RTP research.[^42^, ^43^, ^49^] However, to date, cellulose‐based RTP materials still lack a systematic and comprehensive overview in terms of emission mechanism elucidation and construction strategies.

This review provides a comprehensive overview of recent advances in RTP materials based on cellulose and its derivatives. First, the CTE mechanism underlying cellulose‐based nonconventional luminophores is introduced. Subsequently, construction strategies for cellulose‐based RTP materials are systematically discussed from four aspects: regulation of aggregation structures, reconstruction of clustered emission centers, functionalization of hydroxyl groups, and host‐guest doping strategies. Finally, representative applications in information security, bioimaging, and environmental monitoring are summarized (Figure 1). This review aims to deepen the understanding of the intrinsic mechanisms governing RTP materials derived from cellulose and other nonconventional biomass resources, and to provide valuable theoretical insights and strategic guidance for the rational design and development of long‐lifetime RTP materials in the future.

**FIGURE 1:**
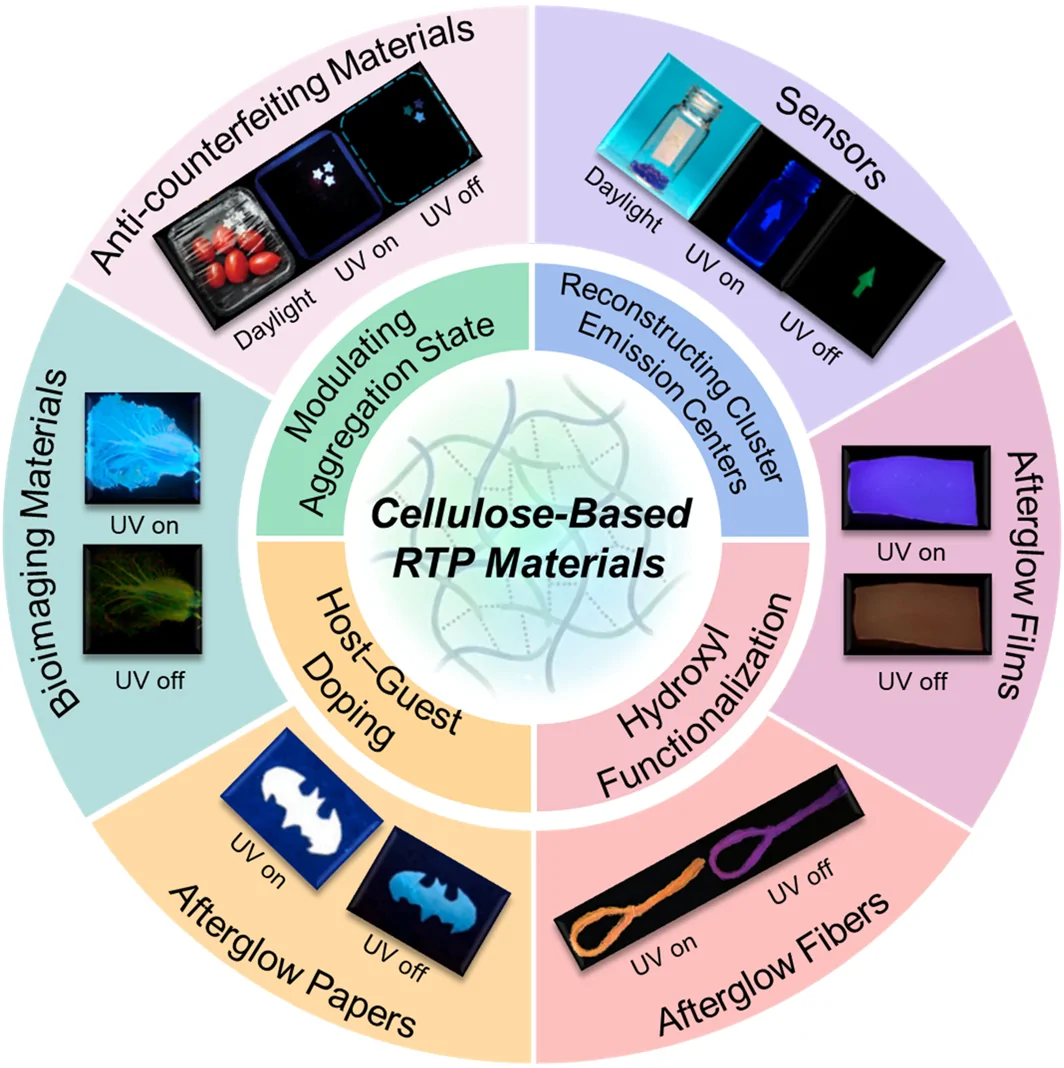
Construction strategies and applications of cellulose‐based room temperature phosphorescent materials.[^21^, ^23^, ^42^, ^43^, ^44^, ^45^]

## 2 THE EMISSION MECHANISM OF CELLULOSE‐BASED RTP MATERIALS

Small‐molecule and polymeric organic luminescent materials that contain elements with lone‐pair electrons (such as N, O, or S), multiple bonds (C=C, C ≡ C), or other functional groups but lack extensive conjugated structures are referred to as nonconventional luminophores, including cellulose,^[50]^ bovine serum albumin,^[51]^ lysine,^[52]^ and boric acid.^[53]^ To explain these emission phenomena, various mechanisms have been proposed, including oxidation,^[54]^ carbonyl aggregation,^[55]^ H‐bond formation,^[56]^ electron delocalization,^[57]^ and end‐group effects.^[58]^ However, these mechanisms lack interconnectivity and fail to account for the diverse behaviors observed in nonconventional RTP systems. In 2013, Yuan et al. proposed the CTE mechanism.^[39]^ In this mechanism, nonconventional luminophores containing electron‐rich heteroatoms and unsaturated bonds (such as C=O, C=C, and C=N) can aggregate closely to form effective through‐space conjugation (TSC).^[59]^ This interaction leads to the formation of “clustered chromophores” with increased energy levels and reduced band gaps. This phenomenon represents the fundamental origin of the intrinsic emission observed in nonconventional emissive materials.^[52]^

In the monomolecular state, nonconventional luminophores possess a large energy gap (Δ*E*) between the ground and excited states. Consequently, their absorption and excitation wavelengths are short, and the emission typically appears in the ultraviolet region. As molecular aggregation increases, numerous electron‐rich emissive clusters form within the system.^[60]^ The extensive through‐space electron delocalization within these clusters generates denser energy levels and narrows the energy gap (ΔE_ST_), enabling the absorption of UV and even visible light, followed by red‐shifted emission extending into the visible and near‐infrared regions. As the energy gap between the singlet and triplet excited states decreases, the intersystem crossing (ISC) process becomes more feasible.[^39^, ^61^]

Specifically, the clustering of oxygen atoms in cellulose and the overlap of their lone‐pair electron clouds within clustered chromophores (through‐space electron delocalization) produce an effect similar to that of electron delocalization in conventional covalent conjugation systems. This delocalization extends the effective conjugation length and enhances molecular rigidity, thereby facilitating excited‐state emission. Meanwhile, the system contains abundant H‐bonds, van der Waals forces, dipole‐dipole interactions, and polymer chain entanglements, all of which create effective intra‐ and intermolecular interactions. These interactions promote further conformational rigidification of the emissive clusters, effectively suppress non‐radiative transitions, and enhance emission efficiency (Figure 2).^[62]^ In addition, introducing organic or inorganic small molecules or ions through different construction strategies can further promote the ISC process and suppress non‐radiative decay, thereby achieving efficient phosphorescence emission.[^63^, ^64^, ^65^, ^66^] For example, Zhou et al.^[67]^ prepared CNCs in three aggregated states (tablet, powder, and film) based on the density‐promoted emission (PDE) strategy. By precisely regulating the oxygen atom spacing in CNCs, they effectively tuned cluster emission centers with different luminescence capabilities, thereby realizing the efficient modulation of RTP. Theoretical calculations revealed that the SOC values from T_1_ to S_0_ followed the order of tablet > powder > film. The stronger SOC capability favored the generation of more triplet excitons in tablet CNCs, thus promoting RTP. Similarly, Peng et al.^[68]^ performed theoretical calculations and analyses on Microcrystalline cellulose (MCC) and its oxidized product dialdehyde cellulose (DAC) to clarify the regulatory mechanism of reconstructing cluster emission centers in MCC on its RTP properties. Compared with MCC, DAC exhibited a significantly enhanced SOC capability, with a SOC constant of 54.83 cm^−1^ for the T_1_ to S_0_ transition. Together with the small ΔE_ST_ (0.765 eV) that resulted in slow emission decay, a long‐lived RTP of 514 ms was finally achieved. It can be seen that the reconstruction of cluster emission centers leads to the generation of new excited‐state energy levels and multiple emission centers, thereby enabling the regulation of RTP performance.

**FIGURE 2:**
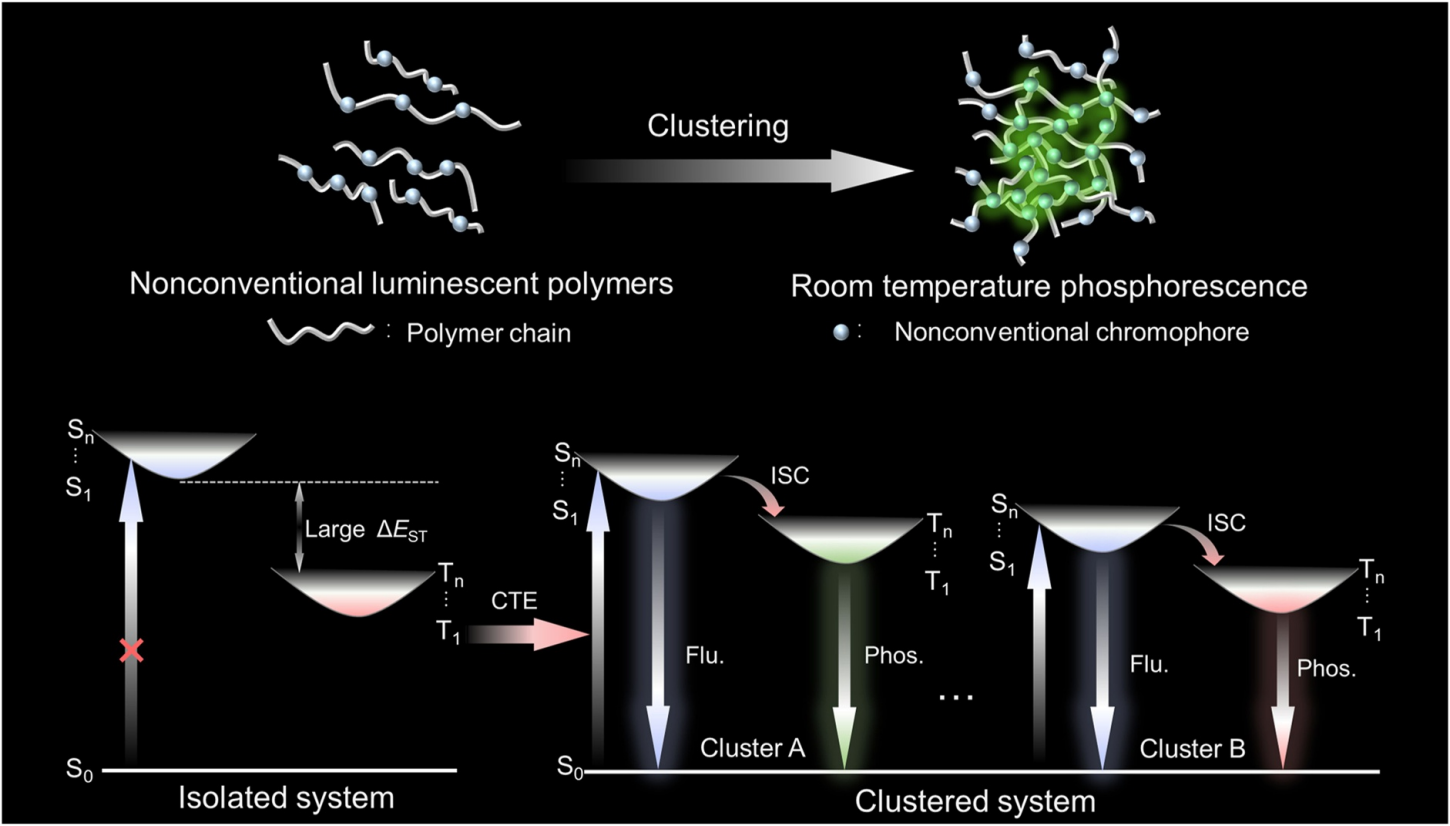
Structure‐property relationships of room temperature phosphorescent emission in nonconventional luminophores^[39]^ and Jablonski diagrams for interpretation of cluster‐triggered emission mechanism.^[62]^

## 3 CONSTRUCTION STRATEGIES OF CELLULOSE‐BASED RTP MATERIALS

Based on the emission mechanism discussed above, the distinct RTP emission behavior observed in cellulose and its derivatives can be reasonably explained. Consequently, researchers have developed various construction strategies to modulate the phosphorescent properties of cellulose‐based RTP materials. As a polymer, cellulose exhibits aggregation‐dependent structures that strongly influence the oxygen‐cluster phosphorescent centers, making the study of this effect highly significant. According to the CTE mechanism, RTP emission can be tuned by reconstructing the oxygen‐cluster emissive centers. Moreover, numerous hydroxyl groups along the cellulose chains can be functionalized with small molecules that promote the ISC process, forming stronger interactions to enhance the RTP emission. Additionally, doping different types of luminescent guests into the cellulose matrix allows the extensive H‐bonding network to provide a rigid microenvironment, further facilitating RTP emission. The following section summarizes recent advances in four different construction strategies.

### 3.1 Modulating the aggregation state of cellulose

As early as 2013, Tang et al. investigated the photoluminescence behavior of various natural products in both solution and crystalline states, and discovered the phenomenon of crystal‐induced phosphorescence (CIP) in natural polymers.^[50]^ Native cellulose exhibits intrinsic RTP characteristics. According to the CIP and CTE theories, variations in its crystallinity and crystal form can modify the structure of emissive clusters. Therefore, tuning the aggregation state of cellulose provides an effective approach to regulate its RTP performance.

MCC, characterized by its high purity and crystallinity, offers facile control over its crystal structure and serves as an ideal model for cellulose‐based studies. In 2019, Du et al. reported that MCC exhibits dual emission characteristics of fluorescence and RTP with excitation dependence, indicating the presence of multiple emissive centers within the MCC structure.^[69]^ Subsequently, Zhou et al. achieved precise control over the crystallinity of MCC and the phase transition from cellulose I to cellulose II through alkali treatment (Figure 3a,b).^[70]^ It was found that variations in the crystallinity and crystal form of MCC alter the RTP emission centers. Higher crystallinity enhances the emission intensity, QY, and lifetime of the samples, particularly strengthening the emission peak at 422 nm. When the difference in crystallinity is minor, the cellulose I polymorph appears to favor the emission of MCC more than the cellulose II polymorph (Figure 3c,d).

**FIGURE 3:**
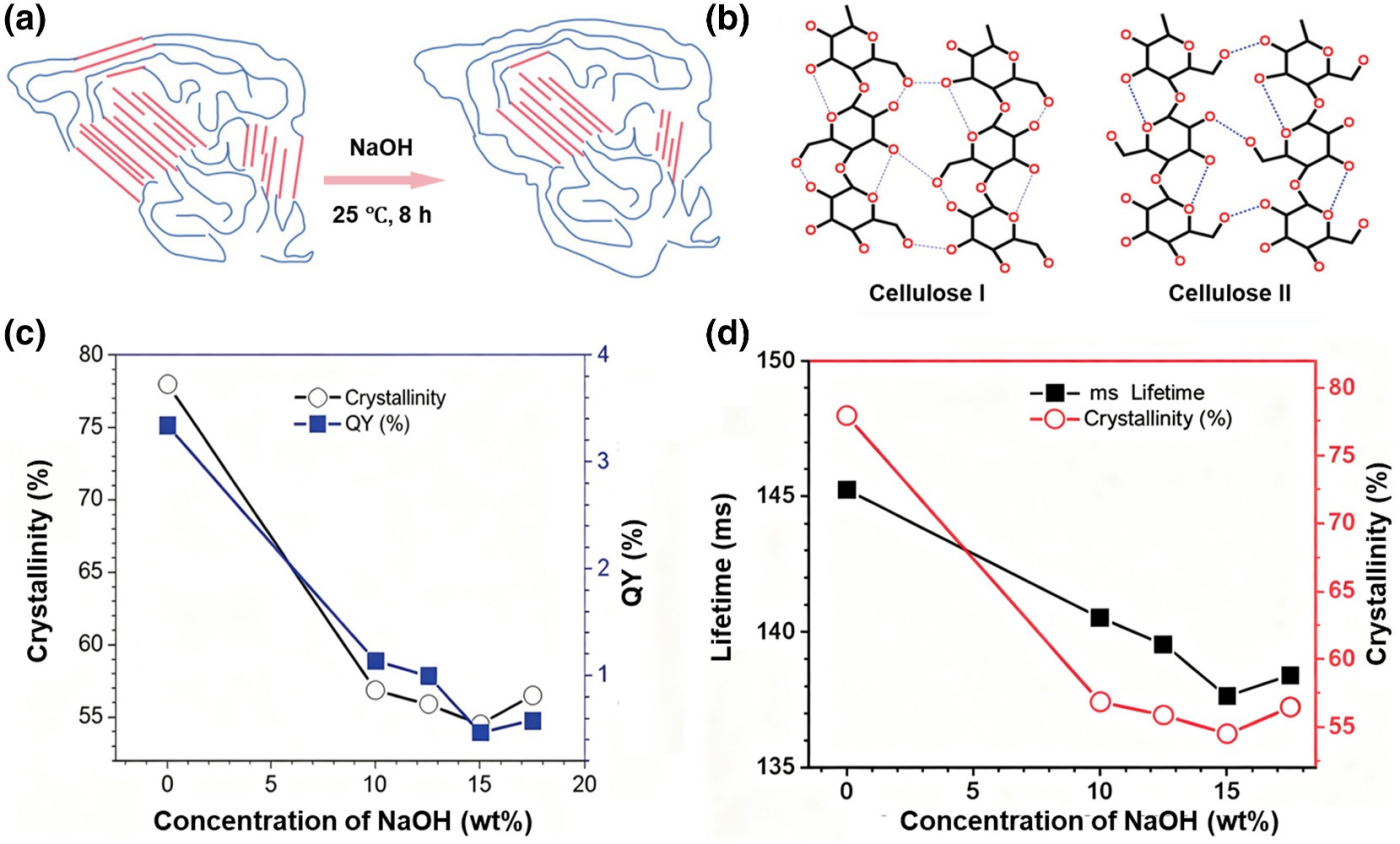
(a) Schematic diagram of crystallinity reduction after NaOH treatment. (b) Schematic diagram of O…O interaction between cellulose I and II hydroxyl groups. (c) The crystallinity and QY of Microcrystalline cellulose samples treated with different concentrations of NaOH. (d) The crystallinity and lifetime of MCC samples treated with different concentrations of NaOH.^[69]^ Copyright 2019, Springer nature limited.

In addition to the effects of crystallinity and crystal form, numerous studies have demonstrated that the tablet‐pressing method is an effective approach to enhance the photoluminescence of unconventional luminophores, particularly their RTP emission. Accordingly, Zhou et al. proposed a strategy of packing density‐promoted emission (PDE), achieved by regulating the molecular packing density (MPD).^[67]^ As shown in Figure 4, Zhou et al. removed the amorphous regions from MCC to obtain cellulose nanocrystals (CNCs) with higher crystallinity and purity. The CNC film, powder, and tablet exhibited progressively increased crystallinity and density, accompanied by a gradual reduction in interplanar spacing. This indicates an enhancement in the MPD of the CNC crystalline regions, leading to changes in the cluster emission centers and improved emission performance. Similarly, Zhu et al.^[45]^ found that removing crystalline water from cellulose enhances molecular packing compactness, suppresses non‐radiative transitions, and stabilizes hydroxyl‐based clusters along the polysaccharide chains, thereby significantly improving its phosphorescent performance with a lifetime reaching the sub‐second range (Figure 5). The dehydration‐induced RTP emission enhancement was observed not only in pure cellulose but also in cellulose‐containing materials such as paper, cotton, and various plant tissues. This finding suggests that cellulose‐based RTP materials can be obtained simply by removing excess moisture without the need for chemical purification.

**FIGURE 4:**
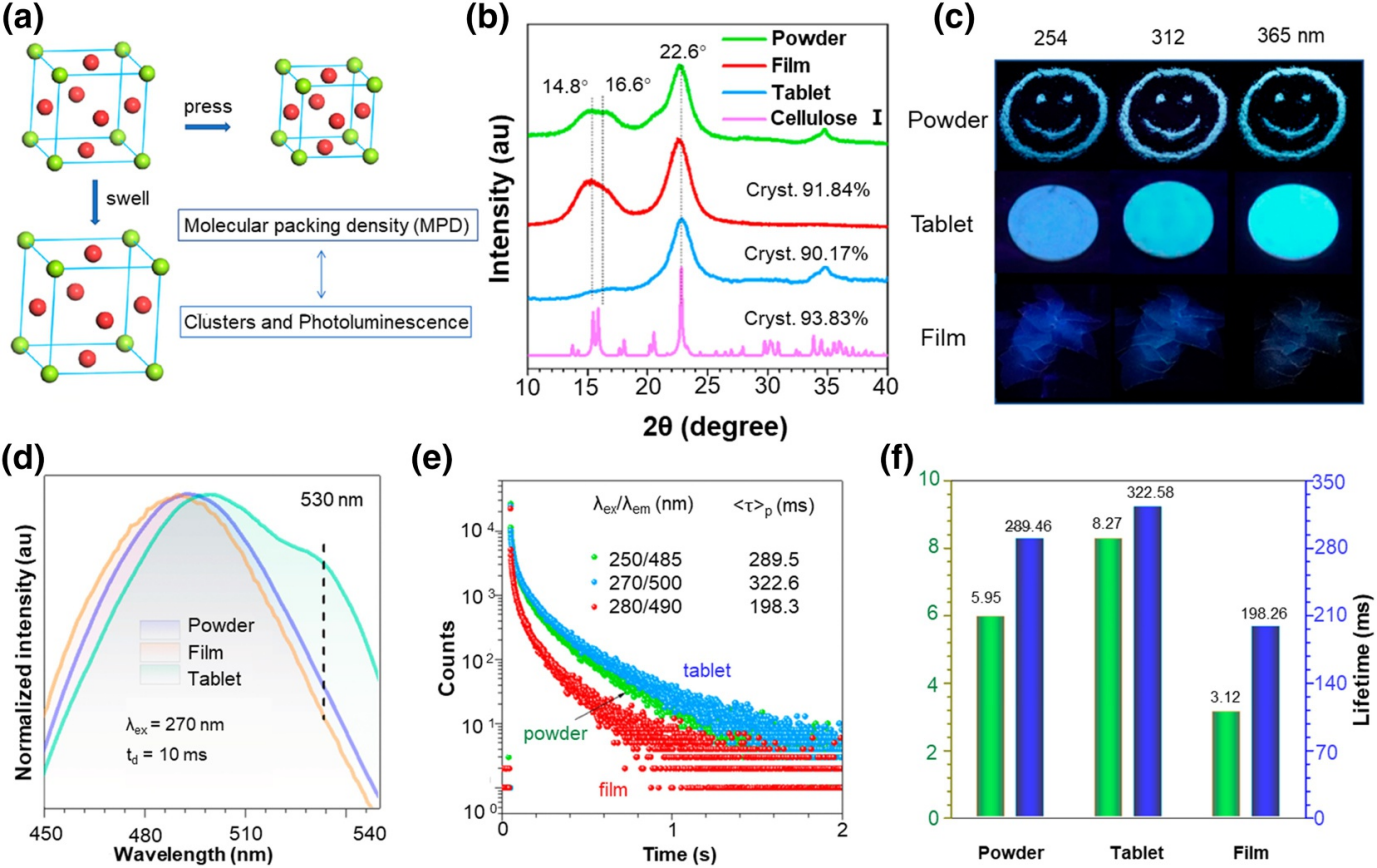
(a) Compression and stretching of atoms in a unit cell affects molecular packing density, cluster formation and photoluminescence. (b) As‐prepared powder, tablet, film: XRD patterns. (c) Sample photos under varied UV lights (ambient conditions). (d) Room temperature phosphorescent spectra of samples in three different states (*λ*_ex_ = 270 nm, *t*_d_ = 10 ms). (e) RTP lifetime of CNC powder, film and tablet. (f) The QY of CNC powder, film and tablet.^[67]^ Copyright 2022, American chemical society.

**FIGURE 5:**
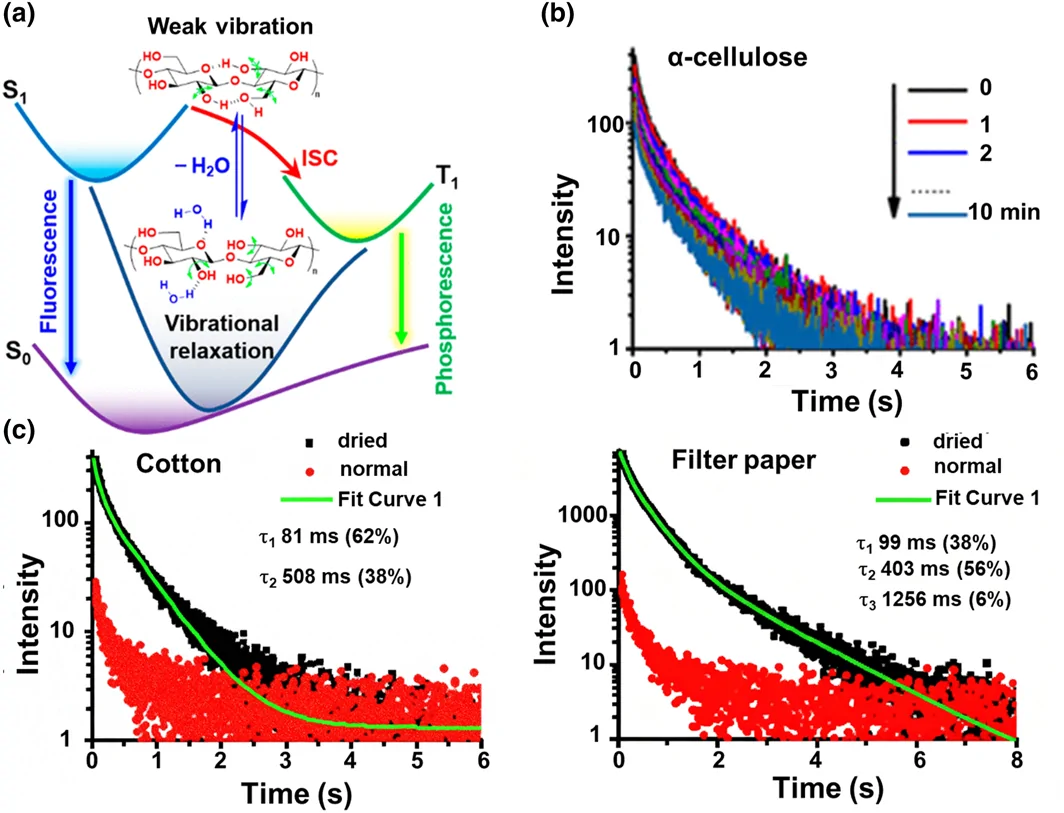
(a) Proposed excited state process of cellulose photoluminescence. (b) Lifetime of dried α‐cellulose (air, RT, 1‐min intervals): *λ*_ex_ = 310 nm, *λ*_em_ = 500 nm. (c) Lifetime of cotton (left) and filter paper (right): before (red) and after (black) RT thermal dehydration (*λ*_ex_ = 310 nm, *λ*_em_ = 500 nm).^[45]^ Copyright 2021, American chemical society.

In addition, controlling the mobility of surface functional groups on cellulose particles can also enhance the RTP performance. As shown in Figure 6, Jin et al.^[71]^ compared the photoluminescence behaviors of CNC foam, CNC film, and CNC/PVA film. They found that CNC foams exhibited the strongest fluorescence intensity and the highest QY (10.94%), but showed the weakest phosphorescence intensity and the shortest phosphorescence lifetime (178.77 ms). Interestingly, the opposite trend was observed in CNC/PVA film, which had the lowest QY (5.15%) but the longest phosphorescence lifetime (569.38 ms). This phenomenon may result from the progressively increased restriction of hydroxyl group mobility on the CNC surface. The formation of additional H‐bonding interactions effectively suppresses the motion of surface hydroxyl groups, thereby enhancing the RTP emission and extending the RTP lifetime. Meanwhile, as the temperature increases from 77 to 298 K, the phosphorescence lifetime of the CNC film decreases, whereas that of the CNC/PVA film remains nearly unchanged. This further confirms that restricting the motion of surface hydroxyl groups on CNC facilitates stronger phosphorescent emission and longer phosphorescence lifetimes.

**FIGURE 6:**
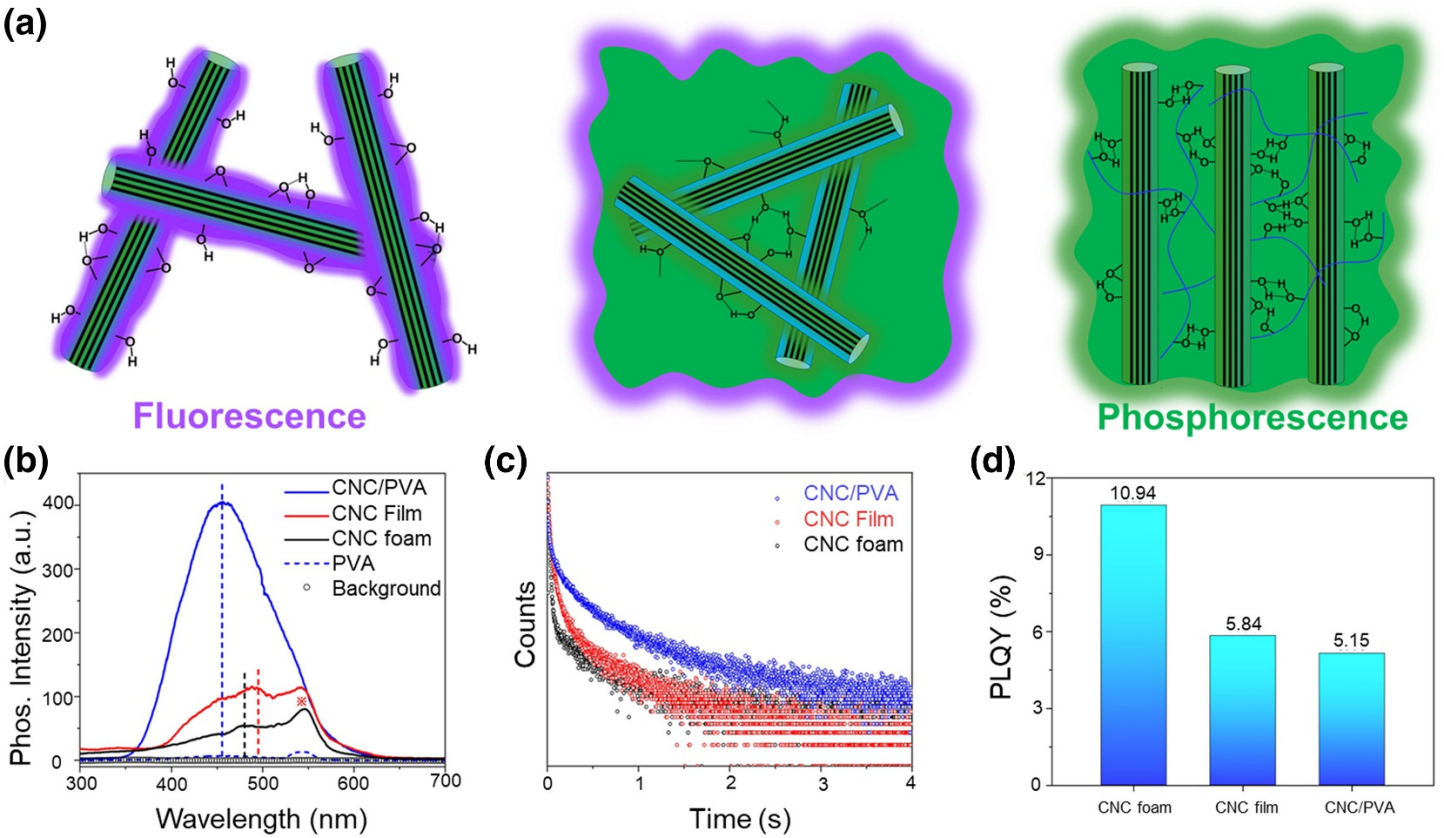
(a) Schematic diagram of H‐bonding interaction in CNC foam, CNC film and CNC/PVA film. (b–d) Room temperature phosphorescent spectra, lifetime at room temperature and PLQY of CNC foam, CNC Film and CNC/PVA film (*λ*_ex_ = 270 nm).^[71]^ Copyright 2023, Springer nature limited.

Therefore, by tuning the crystallinity and crystal form of cellulose—thereby altering the molecular chain conformation and intermolecular interactions—the emission centers of cellulose‐based RTP can be effectively regulated. At the same time, restricting the motion of internal or surface functional groups enhances its RTP emission capability. As the MPD of cellulose increases, electron delocalization becomes more extensive, leading to a more efficient TSC and stronger cluster emission. Moreover, the strengthened intermolecular interactions stabilize the molecular conformations in both ground and excited states, further improving the RTP emission intensity.

### 3.2 Reconstructing the cluster emission centers of cellulose

From a molecular structural perspective, cellulose chains are composed of glucose rings linked by β‐1,4‐glycosidic bonds. Therefore, by modifying the glucose ring structure of cellulose—thus tuning the optically active cluster emission centers—different RTP emissions can be achieved. Peng et al.^[68]^ developed an effective cluster‐reconstruction strategy. They modified the glucose‐ring structure of cellulose chains by stereospecific redox reactions. This approach produced cellulose‐based RTP materials that are long‐lived, processable, and color‐tunable. As shown in Figure 7a, MCC was first oxidized with sodium periodate to yield 2,3‐dialdehyde cellulose (DAC). DAC was then either further oxidized with sodium chlorite to produce 2,3‐dicarboxylic acid cellulose (DCC), or reduced with sodium borohydride to give 2,3‐diol cellulose (reduced DAC, RDAC). The oxygen‐cluster structures and their local environments changed accordingly after the redox treatments. The oxidized products DAC and DCC contain additional unsaturated C=O groups. These groups enable n–π* transitions that enhance SOC and promote the generation of triplet excitons via ISC. As a result, DAC exhibits a distinctive long afterglow. Moreover, increasing the oxidation degree of DAC further increases its phosphorescence lifetime. Upon further oxidation, DCC forms different aggregated states that produce a higher‐energy blue afterglow. In contrast, RDAC, obtained by reduction of DAC, bears exocyclic primary hydroxyl groups. These groups create a more rigid crystalline environment that suppresses non‐radiative relaxation of triplet excitons, yielding an ultralong lifetime of 799 ms (Figure 7b). Boric acid crosslinking enables secondary hardening of RDAC, resulting in a nonconjugated, nonconventional luminophore (DC‐BA) with a prolonged phosphorescence lifetime and a photoluminescence QY of 2.22% (Figure 7c).^[65]^ Moreover, this stepwise hardening strategy generates multiple oxygen‐cluster chromophores within the system, leading to abundant energy levels and reduced energy gaps, thereby producing excitation‐dependent color‐tunable afterglow. This stepwise hardening approach is also applicable to biomass materials such as chitosan and sodium alginate, demonstrating a certain degree of universality. In addition, various arylboronic acids with different conjugation degrees can be anchored onto RDAC through click chemistry in alkaline aqueous solution, leading to the formation of full‐color‐tunable RTP cellulose.^[72]^ The resulting cellulose materials exhibit excellent RTP performance (Figure 7d), with a phosphorescence lifetime up to 2.67 s, a photoluminescence QY of 9.37%, and an absolute afterglow brightness of 348 mcd m^−2^. Therefore, altering the glucose‐ring structure to reconstruct the aggregation of oxygen clusters in cellulose is an effective strategy for tuning its phosphorescent properties.

**FIGURE 7:**
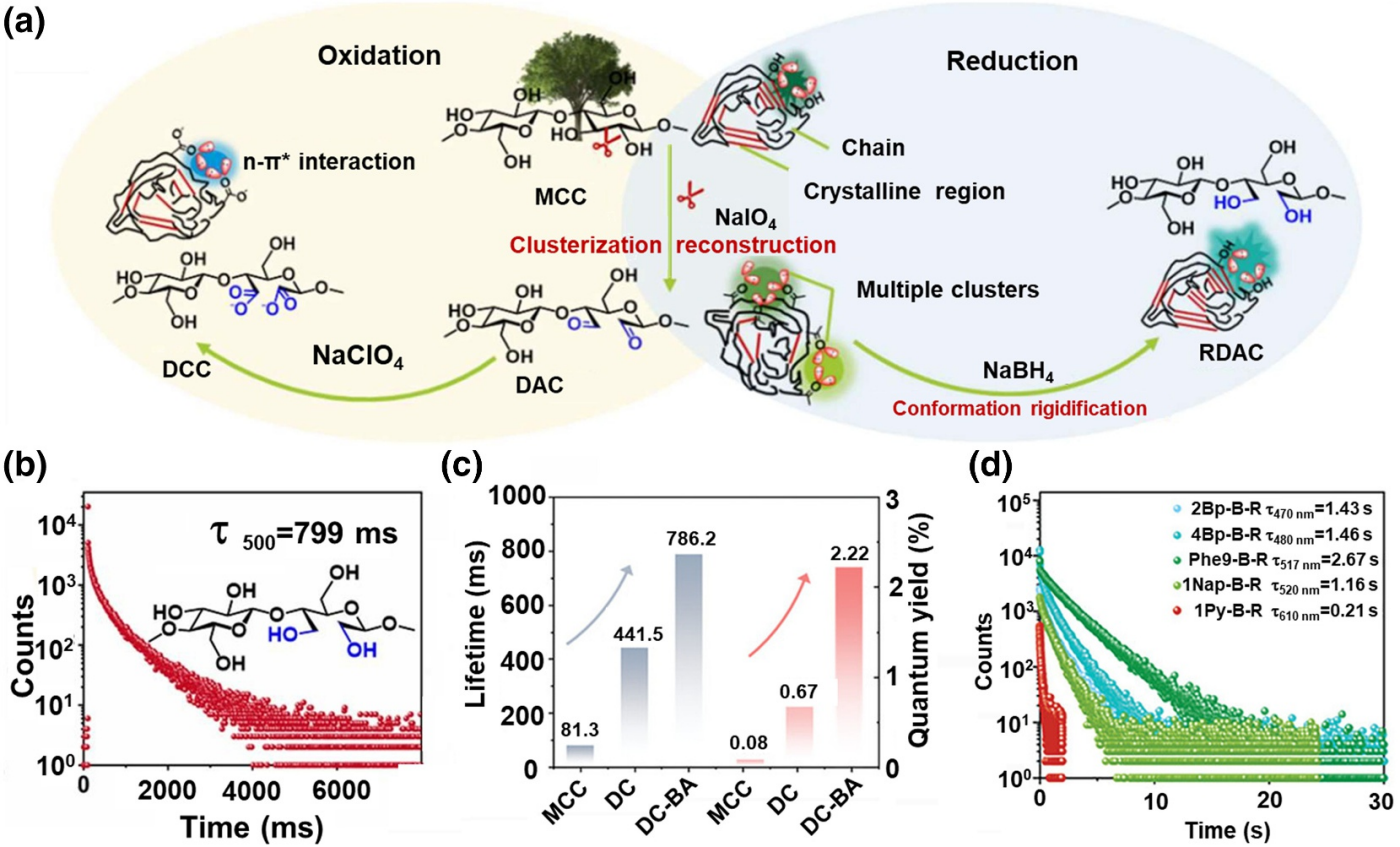
(a) Dialdehyde cellulose, DCC, RDAC synthesis process and cellulose clusterization reconstruction schematic diagram.^[68]^ Copyright 2023, Elsevier B.V. (b) RTP lifetime of RDAC. (c) RTP lifetime and QY for MCC, DC, DC‐BA (*λ*_ex_ = 280 nm).^[65]^ Copyright 2024, John Wiley and Sons. (d) RTP lifetime of the arylboronic acids‐RC.^[72]^ Copyright 2024, John Wiley and Sons. RTP, room temperature phosphorescent.

### 3.3 Hydroxyl functionalization of cellulose

The cellulose molecular chains are rich in hydroxyl groups, which form a strong H‐bonding network. This rigid structure endows cellulose with weak RTP emission. Although chemical modification of hydroxyl groups can disrupt the H‐bonding network, it often results in weakened or even quenched phosphorescence. To enhance the RTP emission, hydroxyl groups can be alkylated, acylated, or ionized while maintaining partial H‐bonding. Introducing π‐conjugated or phosphorescent substituents in place of hydroxyl groups can strengthen SOC or suppress non‐radiative transitions. Therefore, there remains significant potential for the development of cellulose‐based RTP materials.

**Alkylation and acylation modification**: Chemical modification of hydroxyl groups in natural cellulose, such as alkylation and acylation, can produce cellulose derivatives for industrial applications. These derivatives retain the fundamental cellulose backbone while introducing various functional groups. Du et al.^[69]^ investigated the photophysical properties of MCC and three typical cellulose derivatives, including 2‐hydroxyethyl cellulose (HEC), hydroxypropyl cellulose (HPC), and cellulose acetate (CA). As shown in Figure 8, a comparison revealed that the powders of MCC, HEC, and HPC exhibit both fluorescence and RTP emissions, whereas the CA powder shows much weaker emission with no obvious RTP. For the cellulose derivative solutions, the fluorescence intensity of HEC and HPC increases gradually with increasing concentration, showing a concentration‐enhanced emission behavior. In contrast, the emission of the CA solution is very weak. The RTP emissions of these four materials can be attributed to the CTE mechanism, in which the aggregation of n‐electrons from oxygen atoms in ‐O‐, ‐OH, or C=O groups promotes emissive transitions. MCC, HEC, and HPC contain abundant hydroxyl groups that form H‐bonds, enhancing conformational rigidity and promoting close contact among nonconventional chromophores. Moreover, their higher crystallinity compared with CA further contributes to the enhanced emission.

**FIGURE 8:**
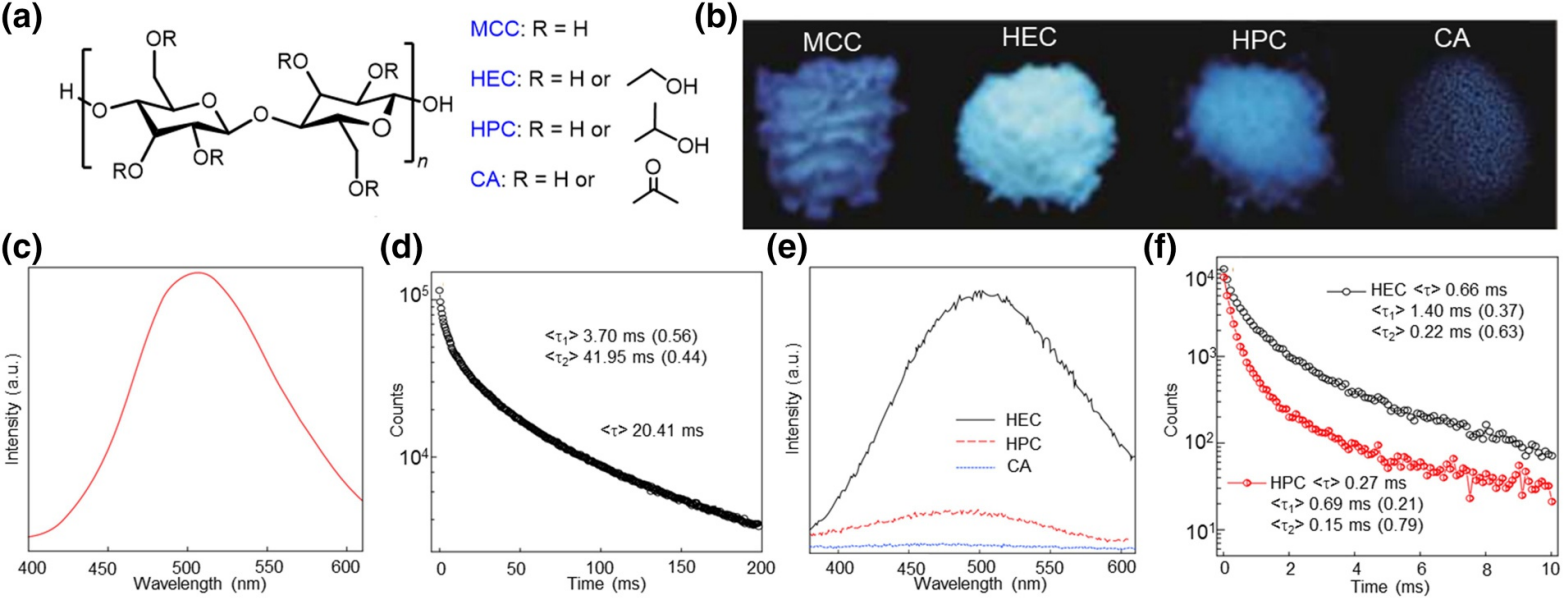
(a) Chemical structures of MCC, HEC, HPC, and CA. (b) Photos of MCC, HEC, HPC, CA powders (365 nm UV light). (c) RTP spectrum (*λ*_ex_ = 330 nm) of MCC powder. (d) Lifetime of MCC powders (*λ*_em_ = 500 nm). (e) RTP spectrum of HEC, HPC, CA powders (RT, *t*_d_ = 1.0 ms, *λ*_ex_ = 330 nm). (f) Lifetime of HEC and HPC powders at R.T.^[69]^ Copyright 2019, Springer nature limited. CA, cellulose acetate; MCC, microcrystalline cellulose; RTP, room temperature phosphorescent.

**Ionization**: As another modification approach, Du et al.^[73]^ introduced Zn^2+^ ions into sodium carboxymethyl cellulose (CMC), a common cellulose derivative, via ion exchange to form an ionic cross‐linked network. The resulting material exhibited efficient and persistent RTP (Figure 9). This behavior can be attributed to two factors. First, the aggregation of carbonyl groups in cellulose promotes the ISC process. Second, the ionic bonds between Zn^2+^ and carboxyl groups, together with H‐bonds involving hydroxyl groups, enhance intermolecular interactions, effectively restricting molecular motion and suppressing non‐radiative transitions, thereby enabling efficient RTP emission. The material achieved a photoluminescence QY of 16.5% and a phosphorescence lifetime of 281 ms.

**FIGURE 9:**
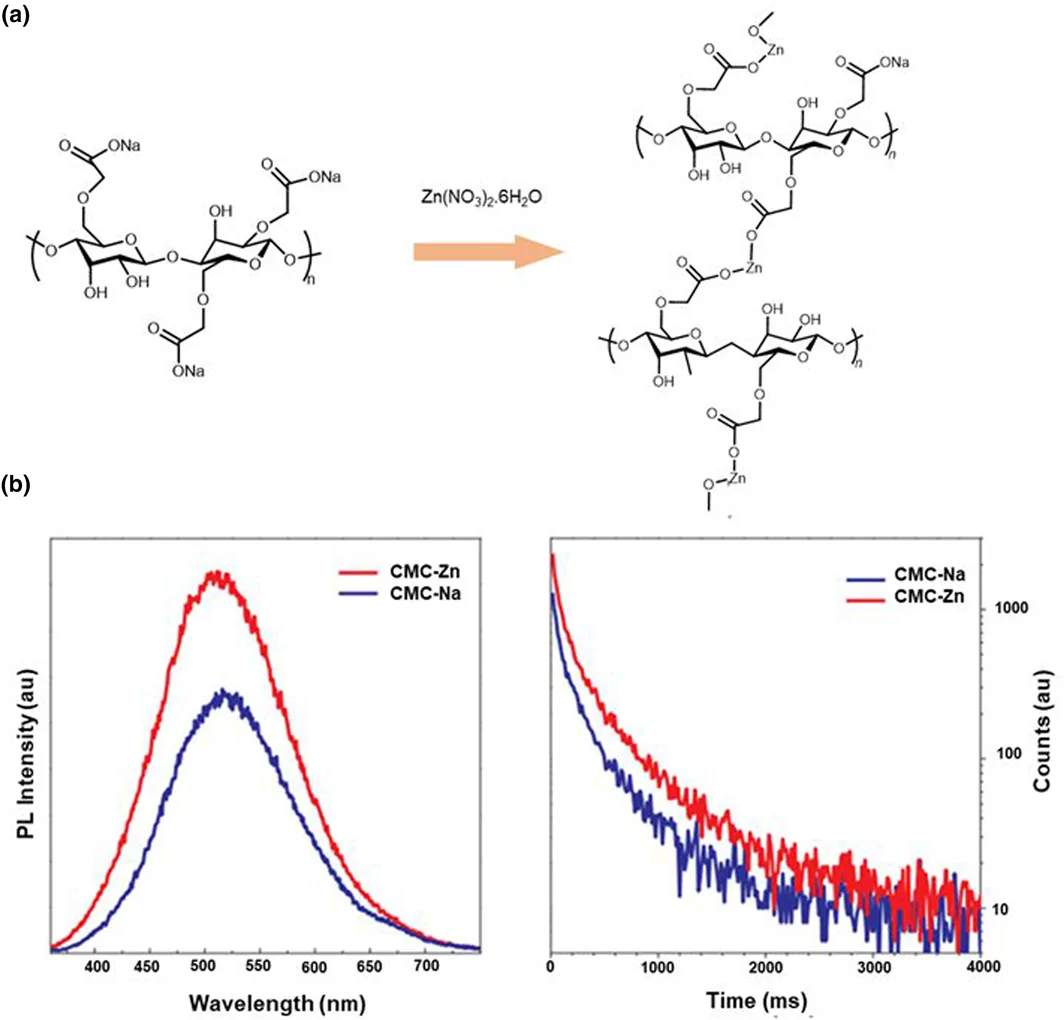
(a) Synthesis route of CMC‐Zn. (b) Room temperature phosphorescent spectrum with *t*_d_ = 1.0 ms and lifetime of CMC‐Na and CMC‐Zn powder at room temperature.^[73]^ Copyright 2018, Elsevier B.V.

Zhang et al.[^23^, ^74^] used natural polymers as raw materials and introduced imidazolium cations into cellulose chains via a homogeneous chemical modification strategy, obtaining a new class of organic phosphorescent materials. The imidazolium cations containing nitrogen heteroatoms effectively promoted the ISC process. The imidazolium cations, counter anions, and residual hydroxyl groups on the cellulose backbone formed multiple H‐bonding and electrostatic interactions, which synergistically suppressed non‐radiative transitions, enabling efficient RTP emission. Experimental results revealed that the degree of substitution, as well as the types of anions and cations, significantly influenced the RTP performance of the materials. As shown in Figure 10a, systematic comparison revealed that shorter and less branched alkyl chains on the imidazolium cation, as well as shorter linker lengths between the imidazolium moiety and the cellulose backbone, were more favorable for phosphorescence emission.^[23]^ Moreover, the type of substituent on the imidazolium cation significantly affected the emission behavior of the materials. The introduction of hydroxyl groups facilitated the formation of additional H‐bonds, thereby suppressing non‐radiative decay, whereas imidazolium cations bearing cyano groups effectively promoted the ISC process.^[23]^ The strong H‐bonding capability of the anion, rather than the heavy‐atom effect, was identified as the dominant factor governing the phosphorescence emission of cationic cellulose derivatives, as strong H‐bonding interactions effectively suppressed non‐radiative transitions (Figure 10b). Furthermore, by introducing a small amount of glutaraldehyde as a crosslinking agent to construct a crosslinked network, the resulting phosphorescent patterns exhibited remarkable antibacterial activity and excellent water resistance.^[74]^

**FIGURE 10:**
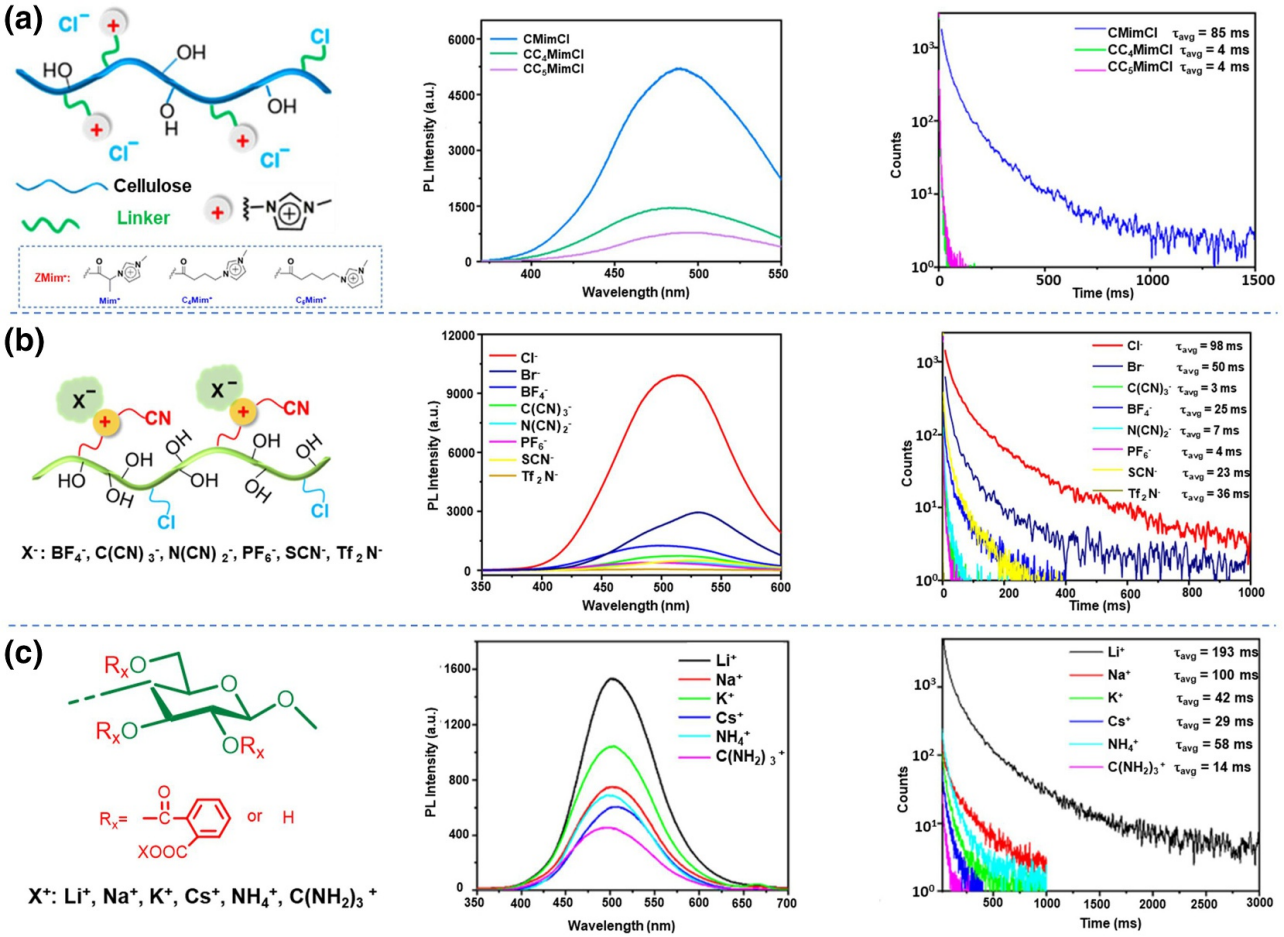
(a) Structures of cellulose samples grafted with different alkyl chain lengths, RTP spectra and lifetime of cellulose samples grafted with different alkyl chain lengths.^[23]^ Copyright 2022, American chemical society. (b) Structures, RTP spectra and lifetime of cellulose samples with different anions.^[74]^ Copyright 2022, Springer nature limited. (c) Structures, RTP spectra and lifetime of cationic cellulose samples (different types).^[75]^ Copyright 2023, Chinese chemical society. RTP, room temperature phosphorescent.

Subsequently, Zhang et al.^[75]^ introduced phenylcarboxylate anionic structures into cellulose chains. This modification not only promoted the ISC process but also enhanced interchain H‐bonding interactions and introduced electrostatic attractions, thereby suppressing non‐radiative transitions and enabling ultralong RTP emission. A smaller cation size, a higher ratio of carboxylate to carboxylic acid groups, an increased number of carboxylic acid substituents on the benzene ring, and an optimal degree of substitution were all found to favor the formation of high‐performance cellulose‐based RTP materials (Figure 10c).

**Substitution with π‐conjugated moieties**: To promote the ISC process, π‐conjugated systems can be introduced to enhance SOC. For instance, π‐conjugated structures can be grafted onto cellulose chains through amide or borate ester linkages. Immobilizing and dispersing these chromophores along the cellulose backbone can effectively prevent aggregation‐induced phosphorescence quenching. Furthermore, the photoluminescence behavior of the material can be tuned by adjusting the molecular aggregation state.

Zeng et al.^[76]^ grafted bulky aromatic derivatives with varying degrees of π‐conjugation onto CMC chains through an amidation reaction, followed by the preparation of membrane materials via a hot‐pressing process. As shown in Figure 11a, the afterglow color of the membranes can be tuned from blue‐green to red, exhibiting photo‐enhanced persistent room‐temperature phosphorescence (p‐RTP) along with good mechanical properties. After UV for 1 min at room temperature, the phosphorescence lifetime of the optimal film increased from 282.1 to 571.1 ms. Theoretical analysis revealed that the abundant intermolecular H‐bonds, spatial packing of aromatic luminophores, and strong π–π interactions are primarily responsible for the excellent photophysical and mechanical performance of the films. Similarly, Gao et al.^[66]^ employed a simple heterogeneous B‐O covalent bonding strategy to graft boronic chromophores with different degrees of π‐conjugation onto the surface of MCC, yielding multicolor RTP cellulose materials with emissions ranging from blue to red. The rigid microenvironment created by the combined B‐O covalent bonds and H‐bonds effectively restricted the thermal motion of the chromophores, stabilized the triplet excitons, and suppressed non‐radiative decay, resulting in an impressive RTP lifetime of up to 1.42 s (Figure 11b). Based on the same principle, the modification of pulp fibers enabled the development of a green and scalable production line for multicolor RTP paper.

**FIGURE 11:**
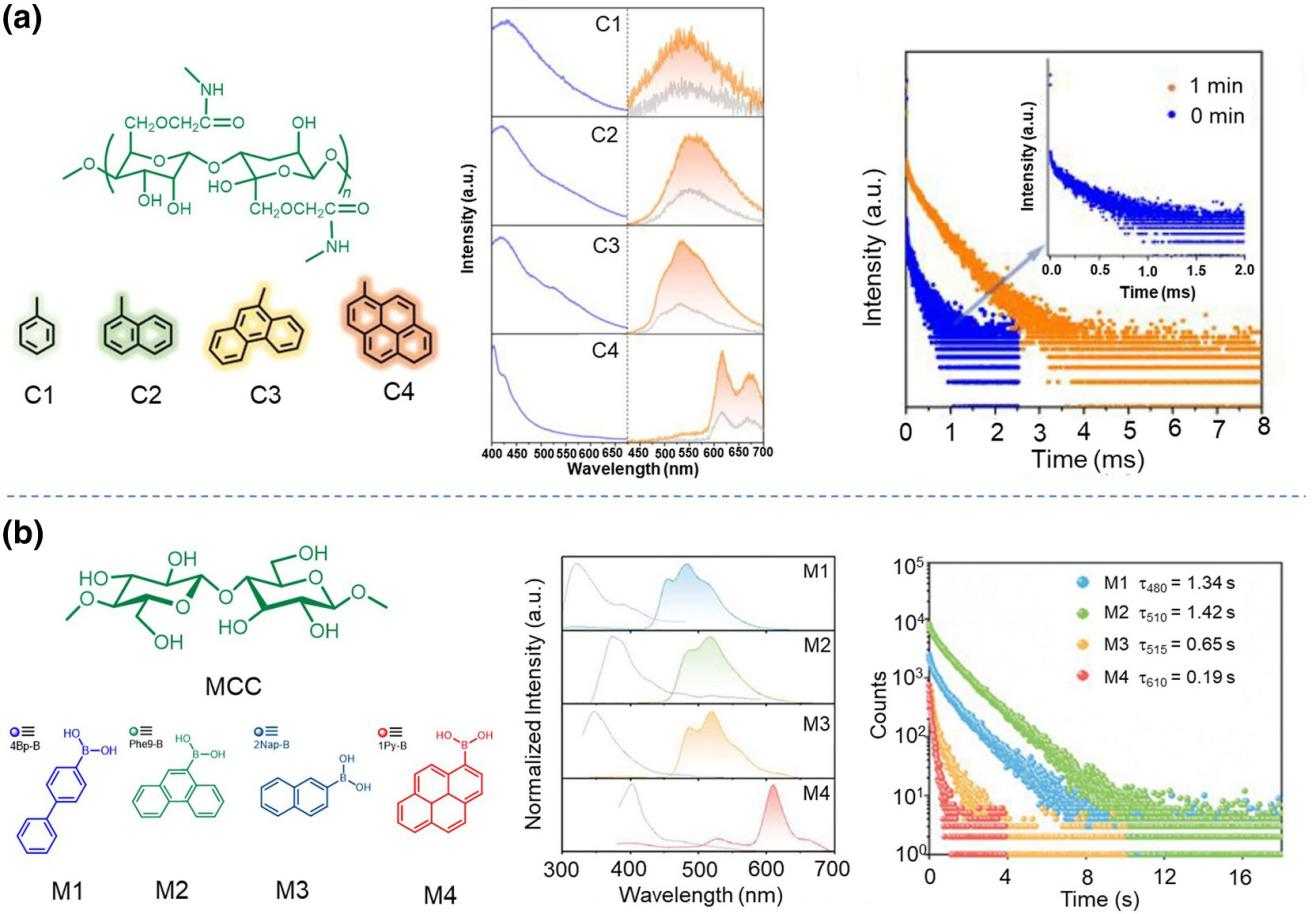
(a) CMC grafted with aromatic rings (various conjugation degrees): structure, phosphorescence spectra and lifetime.^[76]^ Copyright 2022, Elsevier B.V. (b) Microcrystalline cellulose grafted with boronic ester‐containing aromatic rings (various conjugation degrees): structure, phosphorescence spectra and lifetime.^[66]^ Copyright 2023, John Wiley and Sons.

Qi et al.^[77]^ employed the Hantzsch reaction to synthesize a series of cellulose derivatives containing 1,4‐dihydropyridine (DHP) rings as bridging scaffolds, using acetoacetate cellulose (CAA) as the starting material. As shown in Figure 12a, by tuning the structure of aldehyde‐containing aromatic reactants, they obtained RTP materials with excitation‐dependent and color‐tunable emissions and a lifetime of up to 1251 ms. This was attributed to the introduction of acetoacetyl groups in cellulose and the extended conjugation of the DHP ring, which enhanced SOC, thereby facilitating the ISC process and the generation of triplet excitons. Moreover, multiple interactions within the system induced the formation of emissive centers with distinct energy levels and effective conjugation lengths, while simultaneously suppressing non‐radiative transitions. As the degree of conjugation in the DHP structure increased, both the fluorescence and phosphorescence spectra exhibited red‐shifted emissions. Subsequently, Qi et al.^[21]^ developed a scalable method to produce RTP filaments (CAA‐BA) by introducing 4‐aminobenzoic acid luminophores into acetoacetate cellulose (CAA) filaments through a mild enamine reaction. The presence of acetoacetyl and benzoic acid groups promoted the ISC process, whereas multiple H‐bonds provided a rigid microenvironment. As a result, the obtained filaments exhibited a long phosphorescence lifetime of 772 ms and an impressive phosphorescence QY of 45.06% (Figure 12b). Moreover, owing to the significant overlap between the phosphorescence spectrum of the CAA‐BA filament and the absorption spectrum of fluorescent dyes, the afterglow color of the filament could be tuned from blue to green‐yellow and further to rose‐red through an efficient Förster resonance energy transfer (FRET) process.

**FIGURE 12:**
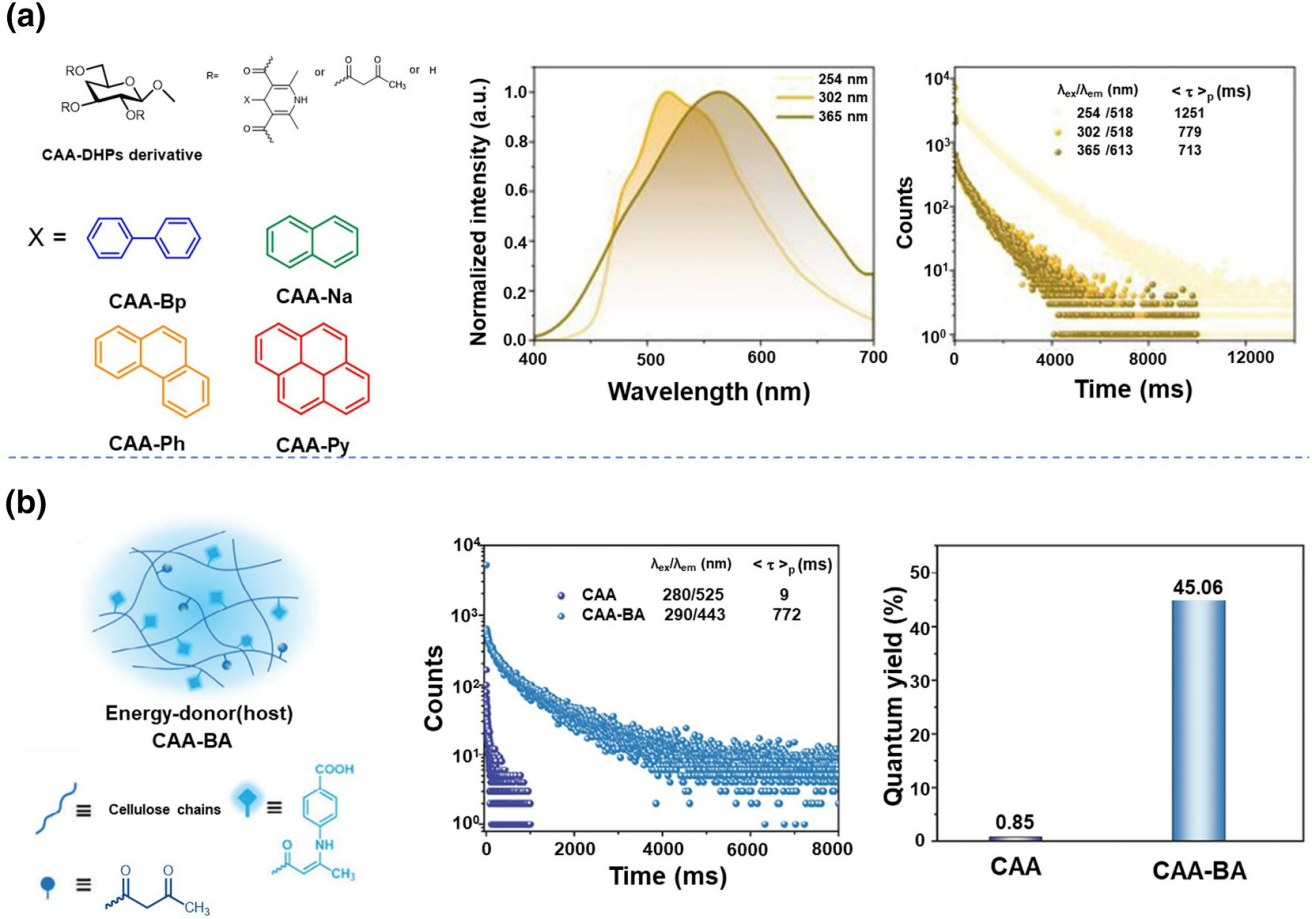
(a) CAA‐DHPs: structures (aromatic rings with various conjugation degrees), room temperature phosphorescent spectra and lifetime (phenanthrene ring‐introduced).^[77]^ Copyright 2023, John Wiley and Sons. (b) Structures, phosphorescence lifetime and QY of CAA‐BA filaments.^[21]^ Copyright 2024, John Wiley and Sons.

In addition to introducing a single π‐conjugated group, Wang et al.^[78]^ grafted complementary cyan and red organic room temperature fluorescent dyes onto CMC chains via amide coupling, developing cellulose‐based RTP materials with tunable multiple color phosphorescence, including white‐light emission. The semi‐rigid cellulose backbone, covalent amide linkages, and dense intermolecular H‐bonds effectively restricted the thermal motion of the fluorescent dyes, suppressed non‐radiative decay, and thereby activated phosphorescence from both dyes under ambient conditions. The resulting materials exhibit excitation‐dependent broad‐spectrum emission and molar ratio‐dependent color tunability. They could also be processed into inks, films, and foams for versatile applications (Figure 13).

**FIGURE 13:**
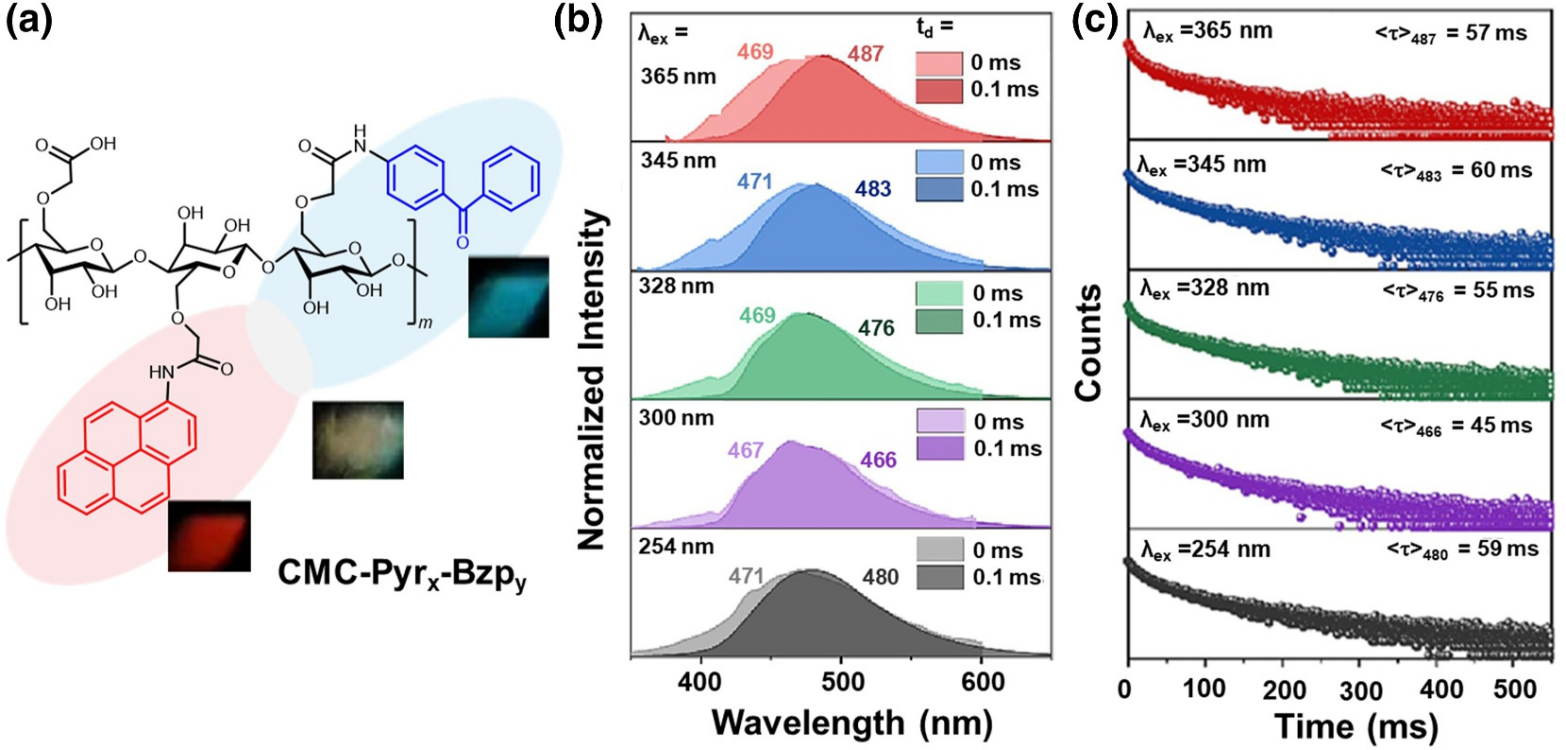
(a) Structures of CMC‐Pyrx‐Bzpy. (b) Normalized PL and room temperature phosphorescent spectra of CMC‐Pyr_0_‐Bzp_1_ (different λ_ex_). (c) Lifetime of CMC‐Pyr_0_‐Bzp_1_ (different λ_ex_).^[78]^ Copyright 2023, Elsevier B.V.

Beyond directly modifying the chemical composition and structure of cellulose, controlling its aggregated state provides an effective strategy to further regulate its emission behavior. You et al.^[79]^ regulated the aggregation state of an anionic cellulose derivative, carboxylated benzene tricarboxylate cellulose (CBtCOONa), by varying the ratio of CBtCOONa to the insoluble inorganic salt CaCO_3_. This strategy yielded ultralong phosphorescent materials featuring multiple emission modes, excitation tunability, and visible‐light‐excited phosphorescence. Experimental results revealed that molecularly dispersed CBtCOONa exhibited blue phosphorescence, while aggregated CBtCOONa emitted green phosphorescence. Samples containing both dispersed chains and aggregates displayed excitation‐dependent, color‐tunable phosphorescence (Figure 14).

**FIGURE 14:**
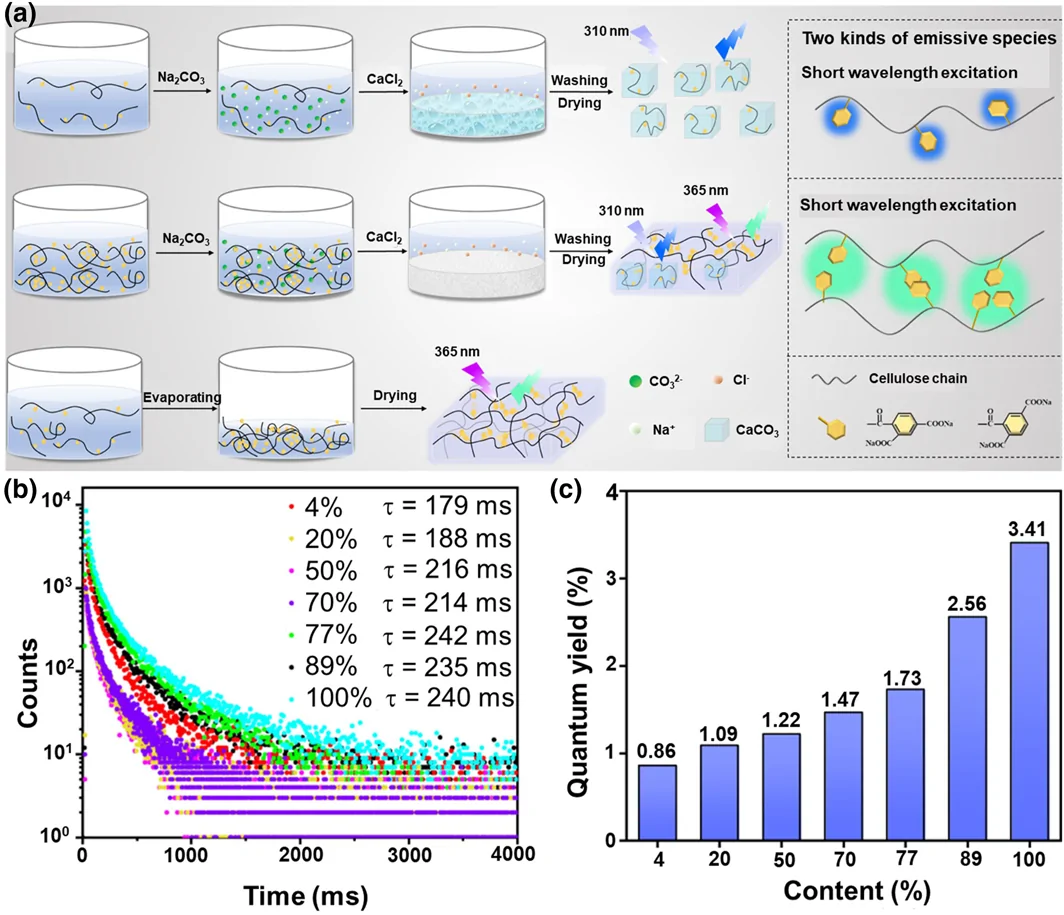
(a) Preparation process and microstructure schematic diagram of the samples. (b) Room temperature phosphorescent (RTP) lifetime of RTP materials (different CBtCOONa contents, *λ*_ex_ = 370 nm, *λ*_em_ = 500 nm). (c) QY of RTP materials with different CBtCOONa contents (*λ*_ex_ = 370 nm).^[79]^ Copyright 2023, Springer nature limited.

Zhang et al.^[80]^ immobilized cellulose derivatives containing aromatic substituents (CX) on the surface of cellulose nanocrystals (CNCs) through H‐bonding interactions. As shown in Figure 15, The resulting nanoscale surface confinement effectively restricted the motion of cellulose chains and luminescent groups, thereby suppressing non‐radiative transitions and enabling efficient RTP emission. The RTP color, lifetime, and QY of the obtained CX@CNC composites could be precisely tuned by introducing CXs with different functional groups, mixing two types of CXs in various ratios, or adjusting the CX/CNC ratio. Consequently, the phosphorescence emission was finely tuned from cyan to red, with a maximum QY of up to 34%.

**FIGURE 15:**
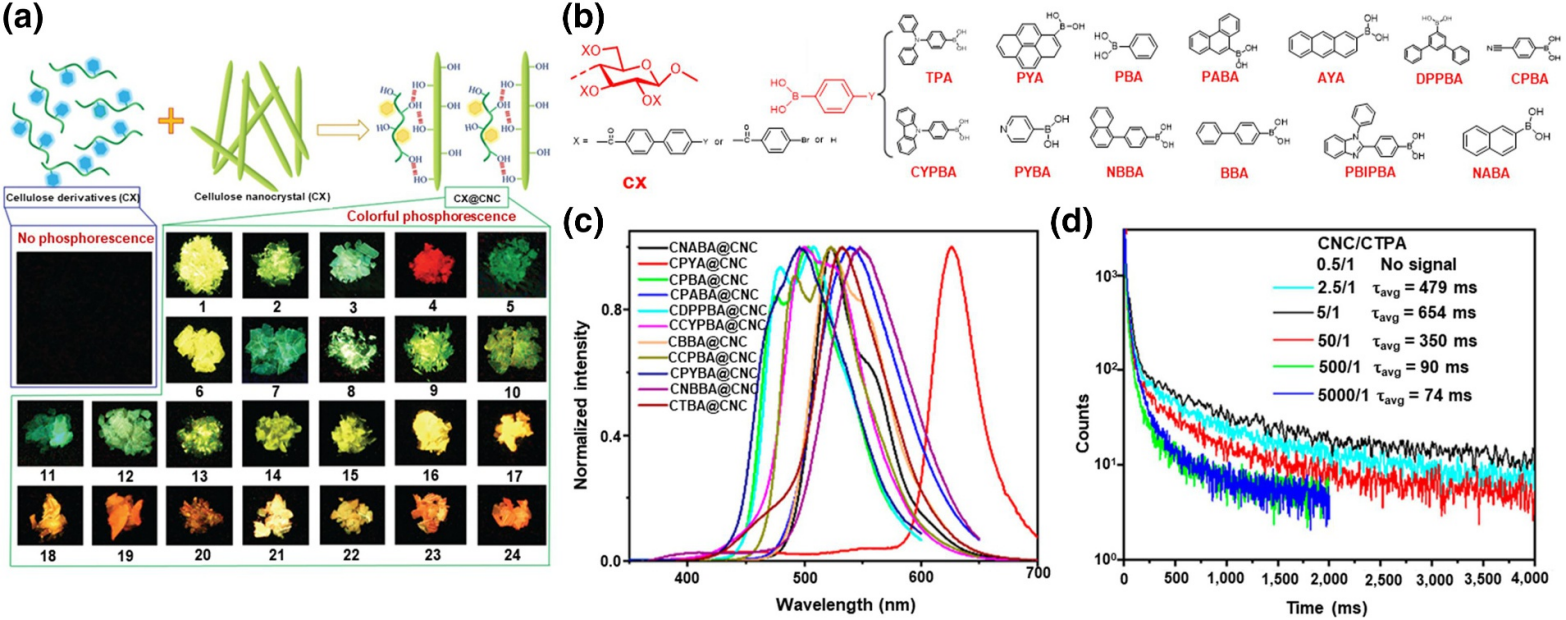
(a) Structural schematic and afterglow photos of cellulose‐based colorful room temperature phosphorescent materials (CX@CNC). (b) The structure of CX. (c) Normalized RTP spectra of CX@CNC. (d) RTP lifetime of CTPA@CNC corresponding to CNC/CTPA with different mass ratios (DS_TPA_ = 0.12).^[80]^ Copyright 2023, American association for the advancement of science.

**Introducing phosphorescent moieties**: Introducing phosphorescent materials with intrinsic long afterglow is also an effective approach to construct cellulose‐based RTP materials. As shown in Figure 16, Yao et al.^[81]^ covalently linked the organic dye fluorescein isothiocyanate (FITC) and the long‐afterglow inorganic phosphor NH_2_‐CaAl_2_O_4_:Eu^2+^,Dy^3+^ (NH_2_‐CAO) onto hydroxypropyl methylcellulose (HMPC) chains, obtaining an organic‐inorganic hybrid cellulose film with pH responsiveness, dual emission, and persistent emission. This work effectively prevented the aggregation‐induced fluorescence quenching of FITC and overcame the limitation of instantaneous photoluminescence in organic dyes. This work broadens the emission wavelength range and extends the emission duration of cellulose‐based fluorescent films, particularly those with environment‐responsive properties, thereby paving the way for novel biodegradable photoluminescent materials.

**FIGURE 16:**
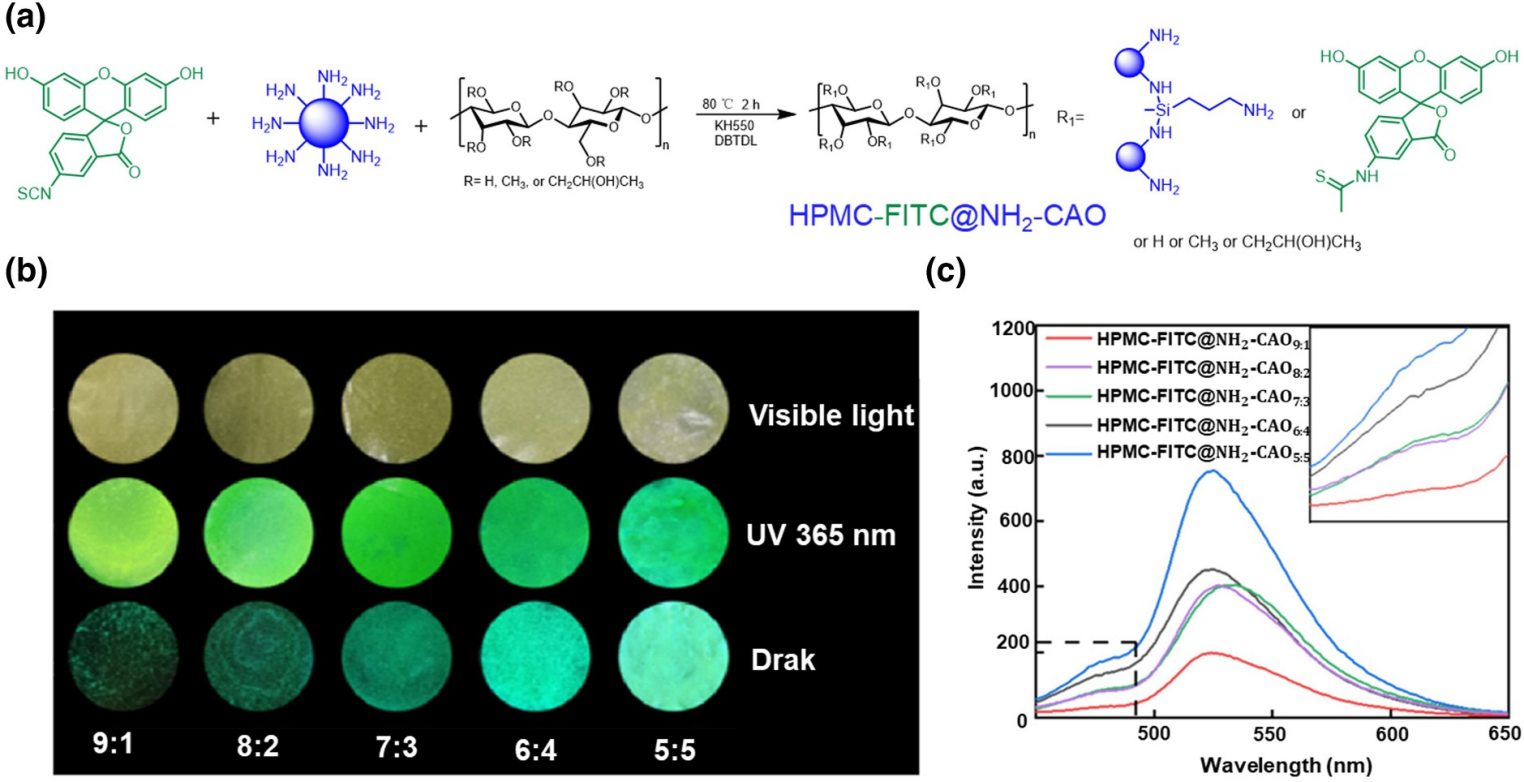
(a) Reaction route of HPMC‐FITC@NH_2_‐CAO Fluorescent Materials. (b) HPMC‐FITC@NH_2_‐CAO films (the DS of FITC = 0.4030) under visible/365 nm UV light/dark. (c) Fluorescence emission spectra of HPMC‐FITC@NH_2_‐CAO (the DS of FITC = 0.4030).^[81]^ Copyright 2021, American chemical society.

### 3.4 Host‐guest doping

In the performance modulation of RTP materials, besides chemical modification, host‐guest doping serves as a crucial pathway: the host provides a stable environment while the guest acts as the luminescent unit, and their cooperation enables efficient RTP emission. The underlying mechanism lies in the host's ability to suppress non‐radiative transitions of the guest and facilitate energy level conversion, thereby enhancing phosphorescence. Various types of guest molecules can be embedded in matrices composed of cellulose and its derivatives. The strong ISC process of small molecules, combined with the rigid network of polymers, contributes to effective RTP emission. Meanwhile, optimizing the doping concentration is also essential for improving photophysical properties.

**Halide salts‐based guests**: Owing to the strong H‐bonding interactions in natural cellulose, chromophores cannot be directly doped into the molecular network of cellulose. Therefore, special strategies are required to open diffusion channels for guest incorporation. Zhu et al.^[82]^ dispersed cellulose in halide salt solutions followed by drying to obtain halide‐doped cellulose (Figure 17). This process induced charge transfer absorption from halide ions to polysaccharide chains and enhanced the ISC process via an external heavy‐atom effect. In addition, the electrostatic interactions between divalent metal ions and the n electrons of cellulose reduced the molecular vibrations of polysaccharide chains, thereby suppressing non‐radiative relaxation. Consequently, enhanced emission of cellulose was achieved without introducing π‐conjugated groups. As the concentration of MgBr_2_ increased, the phosphorescence intensity of cellulose was enhanced and its maximum excitation wavelength exhibited a redshift. Moreover, due to the heavy‐atom effect, the phosphorescence lifetime of bromide‐doped cellulose was shorter than that of chloride‐doped cellulose, and the lifetime decreased with increasing MgBr_2_ concentration. This study provides a new strategy for developing inorganic‐doped cellulose‐based phosphorescent materials.

**FIGURE 17:**
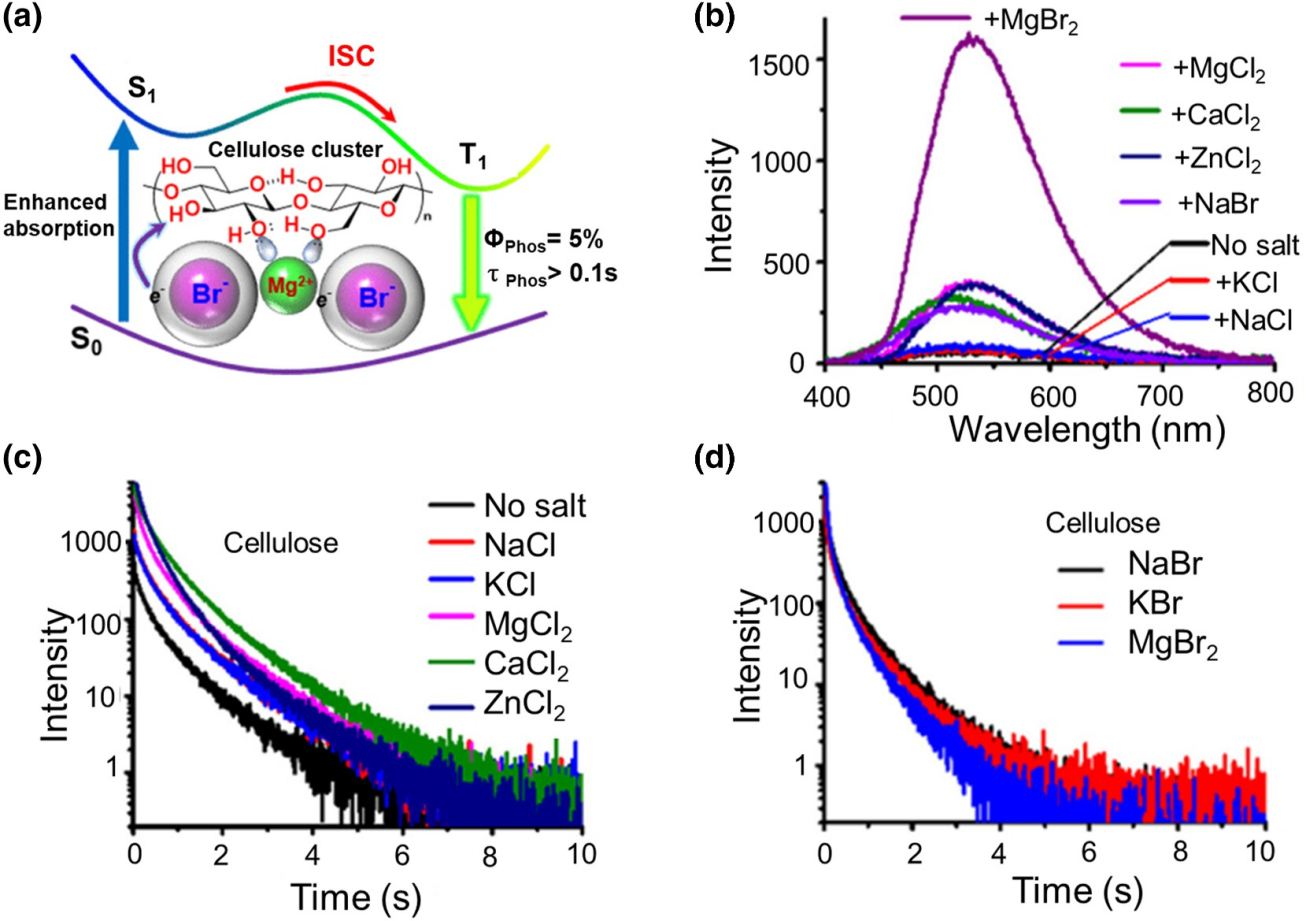
(a) Proposed mechanism: halide salt‐enhanced persistent phosphorescence of cellulose. (b) Room temperature phosphorescent spectra of dried cellulose doped with different salts. (c, d) Lifetime of dried cellulose doped with different salts at R.T. (*λ*_ex_ = 310 nm. *λ*_em_ = 500 nm).^[82]^ Copyright 2022, American chemical society.

**Organic fluorophores‐based guests**: Filter paper, as a cellulose‐based substrate derived from plant fibers, can also serve as a matrix for constructing RTP materials. Guo et al.^[44]^ used potassium tert‐butoxide as a “key” to swell the filter paper, thereby disrupting the dense H‐bonding network of cellulose and enabling the incorporation of indolo[3,2‐b]carbazole derivatives into the matrix, which effectively achieved RTP emission (Figure 18). The cellulose matrix provides a rigid environment and excellent oxygen‐barrier properties, which restrict the molecular motion of the excited states and suppress non‐radiative decay. When a bromine atom was introduced at the ortho‐position of the sulfonyl group in the indolo[3,2‐b]carbazole derivative (ICzS2Br), the intramolecular halogen‐bonding interaction between the halogen‐bond donor (Br atom) and acceptor (sulfonyl group) effectively accelerated the ISC process. As a result, the phosphorescence lifetime of the ICzS2Br‐treated filter paper was prolonged to 1070 ms, with the afterglow persisting for 5 s.

**FIGURE 18:**
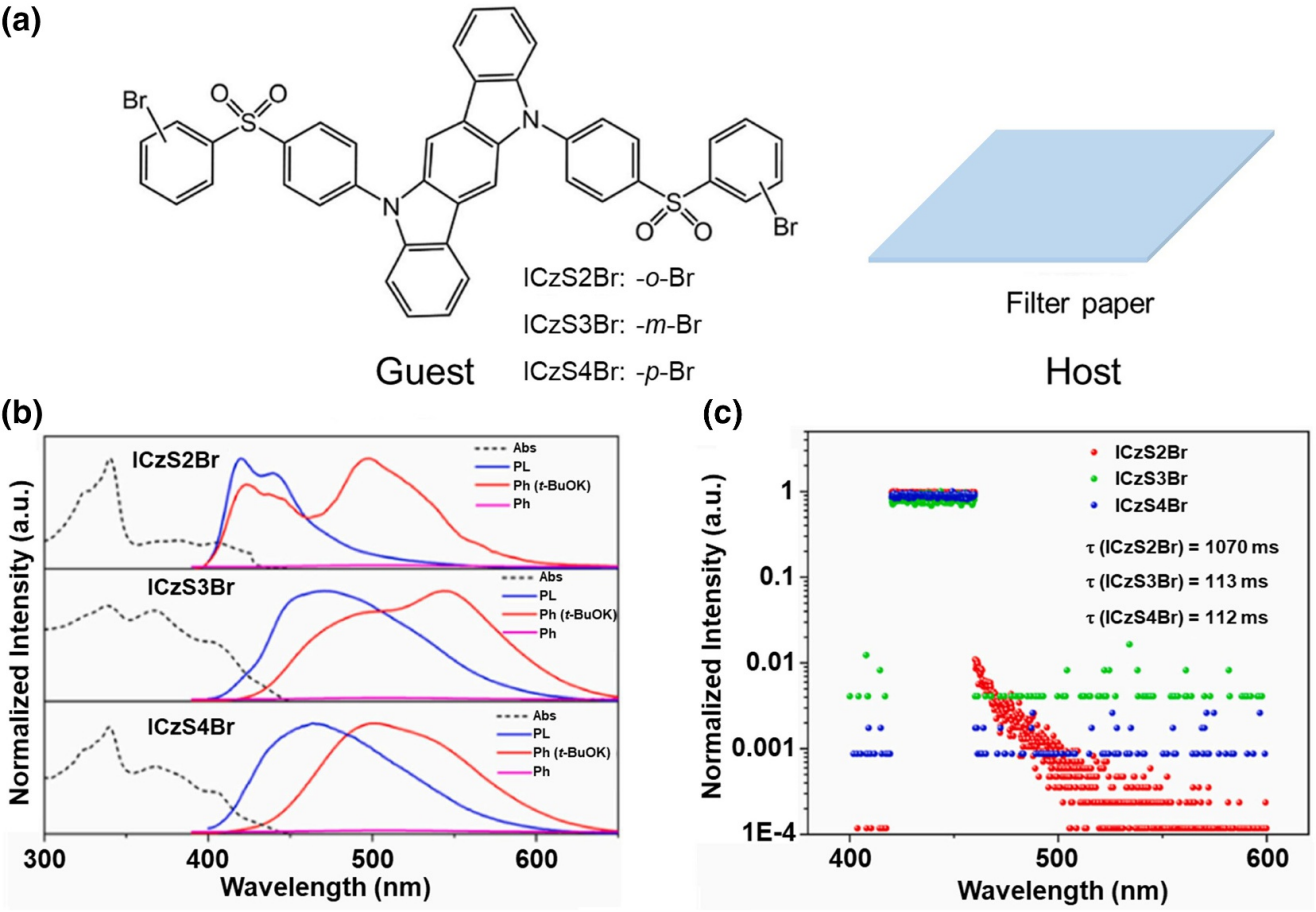
(a) Guest structure and host of the system (b) UV‐vis absorption, fluorescence and phosphorescence spectra of 3‐isomer‐treated filter paper (*λ*_ex_ = 340 nm). (c) Room temperature phosphorescent lifetime curves of ICzS2Br/ICzS3Br/ICzS4Br‐treated filter paper (monitored at 500/545/500 nm, respectively).^[44]^ Copyright 2023, Elsevier B.V.

In addition to plant‐derived cellulose, bacterial cellulose (BC) produced by microorganisms can also serve as a matrix for constructing RTP materials. Nie et al.^[42]^ introduced organic indolo[3,2‐b]carbazole (ICz) isomers into the liquid culture medium during the biosynthesis of BC, allowing the ICz molecules to be directly embedded and immobilized within the BC nanofibers (ICz@BCs) (Figure 19a). The hydroxyl groups along the BC chains form multiple H‐bonds with ICz molecules and create a rigid microenvironment. This rigid network restricts molecular motion, isolates water and oxygen, stabilizes triplet excitons, and prevents their quenching, thereby enabling efficient and long‐lived phosphorescence emission (Figure 19b,c).

**FIGURE 19:**
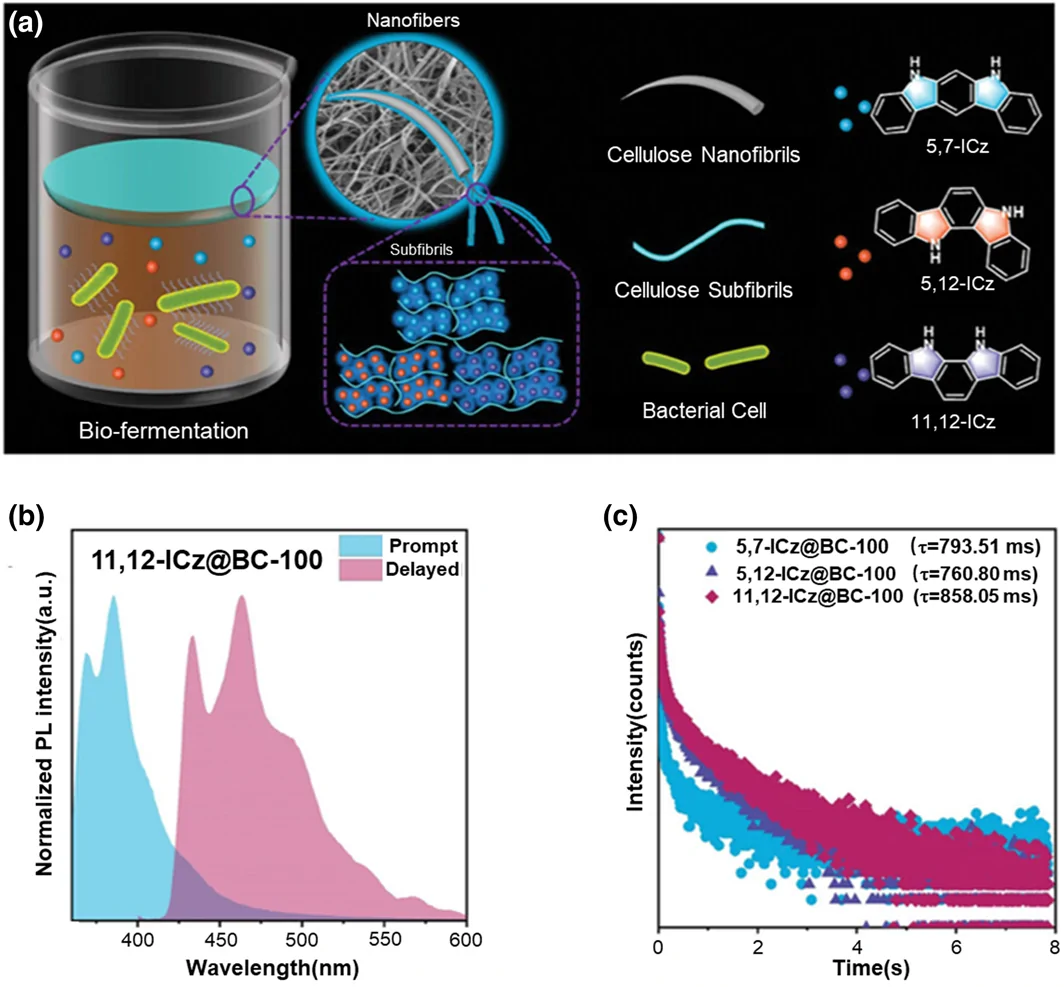
(a) Preparation process of ICz@BC and the molecular structures of ICz (5,7‐ICz, 5,12‐ICz, 11,12‐ICz). (b) Prompt and delayed photoluminescence spectra. (*λ*_ex_ = 328 nm). (c) Room temperature phosphorescent lifetime curves.^[42]^ Copyright 2023, John Wiley and Sons.

As a nanoscale form of cellulose, cellulose nanocrystals (CNCs) possess the unique ability to generate structural colors. Zhou et al.^[43]^ incorporated water‐soluble aromatic sodium salts into CNC dispersions and subsequently cast the mixtures into films. As shown in Figure 20, the resulting films not only retained the unique structural color emission of CNCs but also exhibited tunable persistent phosphorescence that depended on the conjugation degree of the aromatic sodium salts, excitation wavelength, and temperature. This phenomenon originated from the strong ionic and H‐bonding interactions between the aromatic acid molecules and the surface groups of CNCs. These interactions generated multiple clustered emission centers and greatly enhanced the conformational rigidity, which stabilized triplet excitons, promoted the ISC process, and suppressed non‐radiative transitions, leading to p‐RTP emission. Interestingly, at lower temperatures, the cluster emissions originating from cellulose chain segments were more favorable and thus dominated over other emission centers. As the temperature increased, the emissions from other centers dominated by the aromatic sodium salts gradually became predominant, overshadowing those from the cellulose clusters and resulting in temperature‐dependent multicolor phosphorescence.

**FIGURE 20:**
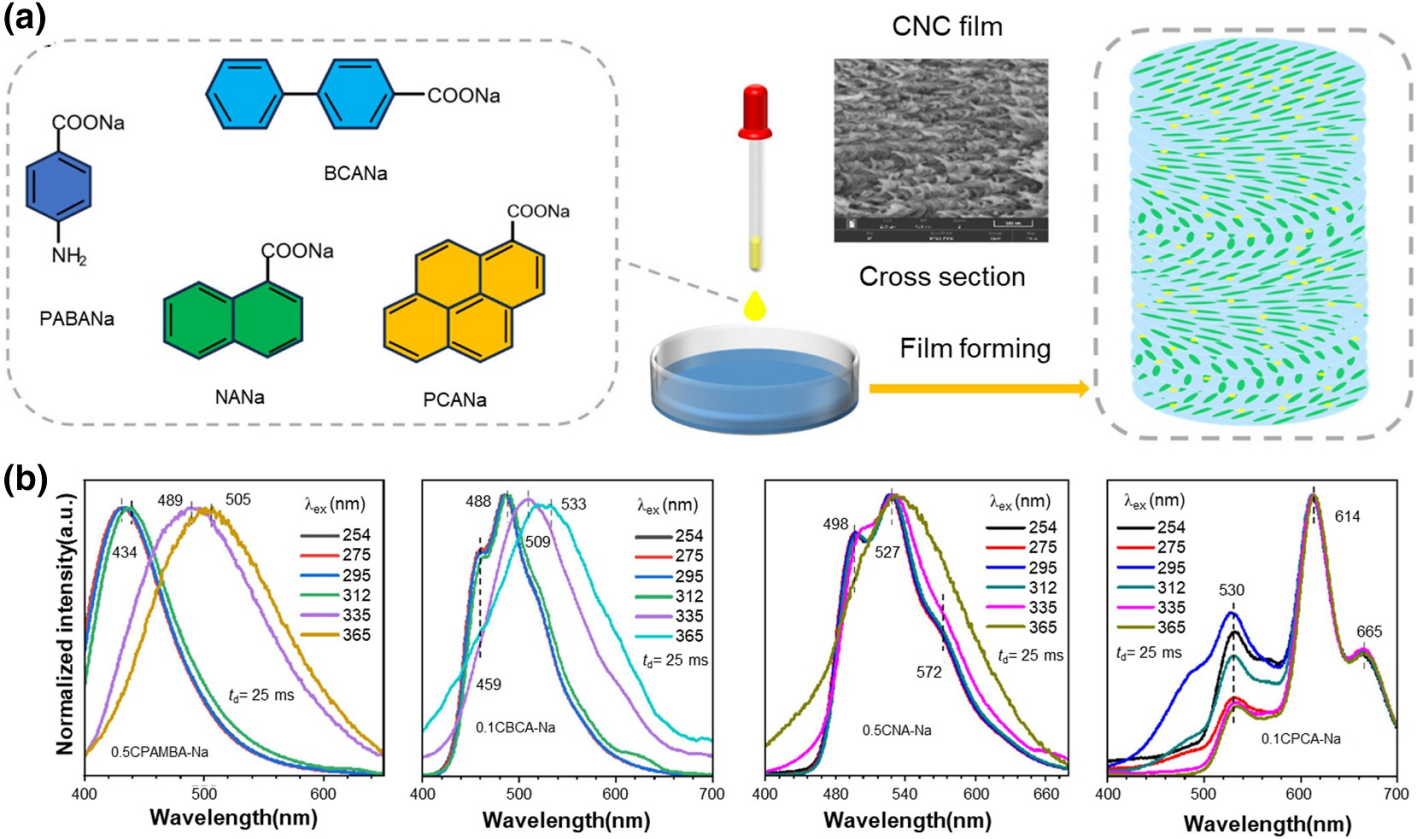
(a) Multicolor p‐RTP film materials: preparation method and structures. (b) RTP spectra of different p‐RTP film materials.^[43]^ Copyright 2024, American chemical society. p‐RTP, persistent room‐temperature phosphorescence.

Moreover, as a naturally chiral nanomaterial, CNC provides a chiral microenvironment through its intrinsic helical structure, which can interact with phosphorescent species to induce circularly polarized room‐temperature phosphorescence (CP‐RTP). Wang et al.^[83]^ utilized the circular dichroism of CNC films and infiltrated PMMA doped with polycyclic aromatic hydrocarbons (naphthalene and pyrene) into the CNC matrix, successfully constructing films that exhibited structural color, phosphorescence, CP‐RTP, and circularly polarized fluorescence (CP‐FL) (Figure 21). In these films, the fluorophores were responsible for both PL and RTP emissions, the polymer matrix provided a rigid environment to promote RTP, and the CNC film acted as a circularly polarized optical filter. By tuning the overlap between the photonic bandgap of the CNC film and the emission spectrum of the fluorophores, the intensity, wavelength, and handedness of both CP‐RTP and CP‐FL emissions could be precisely modulated. The maximum dissymmetry factor (glum‐factor) reached −0.49, and the afterglow duration extended up to 8 s. In addition, dynamic regulation of CP‐RTP and CP‐FL, including on/off switching and positive/negative signal inversion, was achieved by UV that consumed triplet oxygen.

**FIGURE 21:**
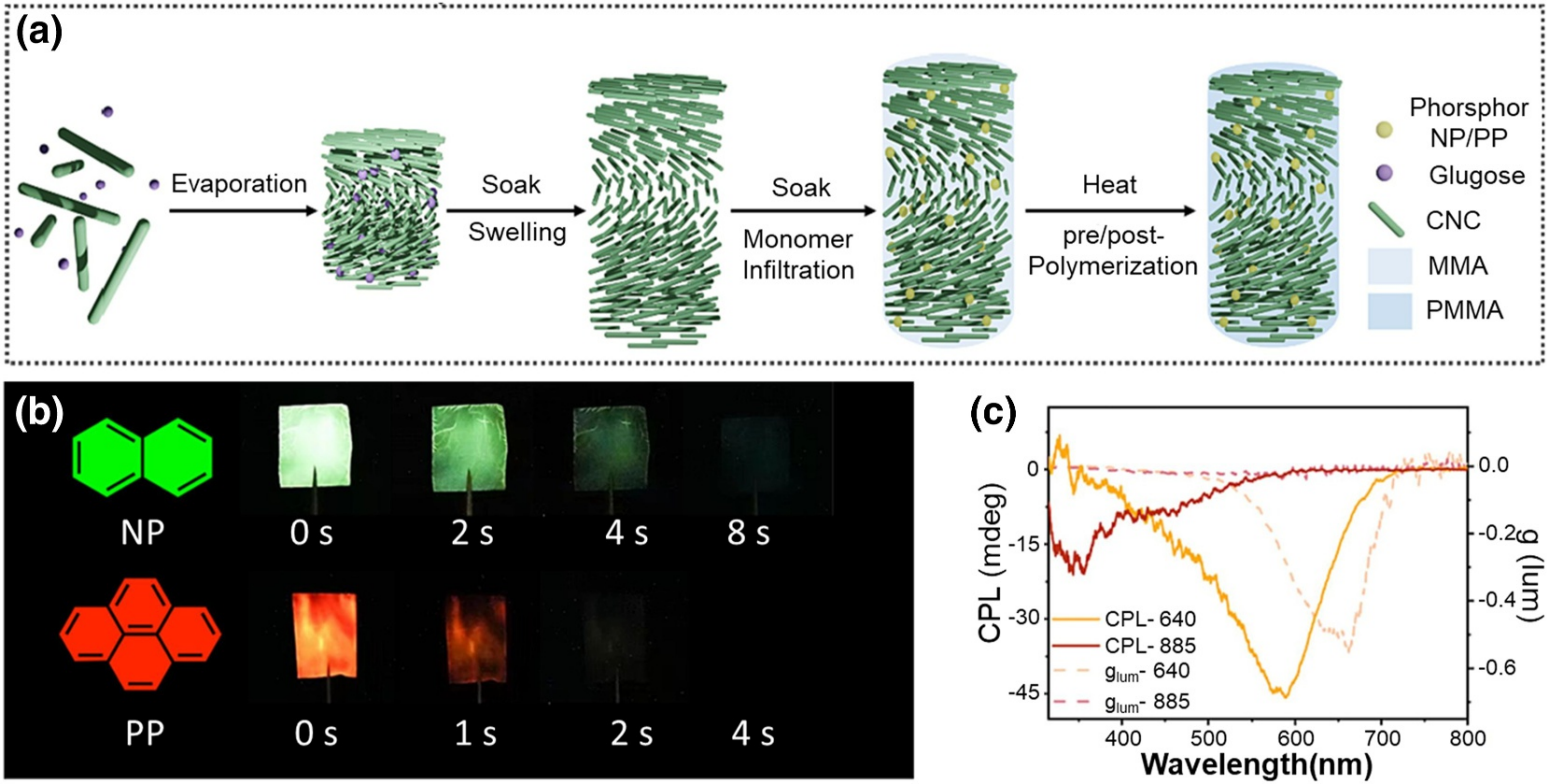
(a) The preparation of CNC‐infiltrated hybrid film (PAH‐doped PMMA). (b) Afterglow photos at different internals after UV. (c) CPL spectra and glum‐factor of NP‐CNC‐640/885.^[83]^ Copyright 2024, Elsevier B.V.

**Carbon quantum dots‐based guests**: Carbon dots (CDs) have attracted considerable attention due to their high QY and tunable emission colors. As shown in Figure 22, Wang et al.^[49]^ dissolved natural cellulose completely in a NaOH/urea aqueous solution and introduced bio‐based CDs, which themselves do not exhibit phosphorescence, into the solution. Upon adding water as a poor solvent, the cellulose precipitated and regenerated, yielding a series of long‐afterglow RTP materials in which CDs were embedded within the cellulose matrix. The resulting materials exhibited RTP lifetimes of up to 190 ms, with emission properties dependent on excitation wavelength, humidity, and temperature. The carbonyl (C=O) groups in the CDs and the strong H‐bonding interactions between the CDs and cellulose chains were identified as key factors promoting phosphorescence generation. In addition, the presence or absence of sodium ions in the samples affected the wavelength of the phosphorescent emission.

**FIGURE 22:**
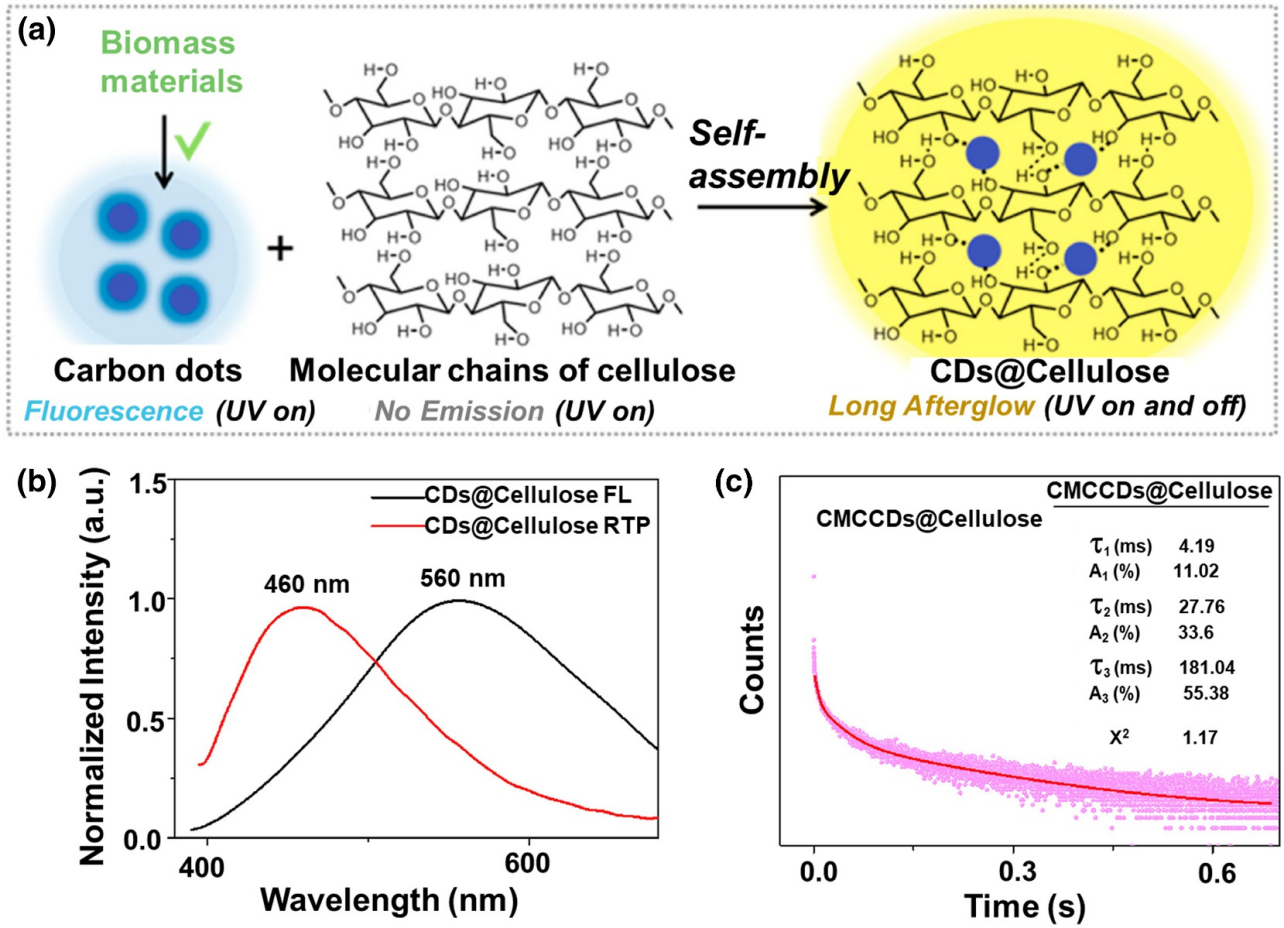
(a) Preparation process of CDs@Cellulose. (b) Fluorescence/phosphorescence spectrum (*λ*_ex_ = 375 nm; red: no delay, black: with delay) of CMC CDs@Cellulose. (c) Phosphorescence lifetime (*λ*_ex_ = 375 nm, *λ*_em_ = 560 nm) of CMC CDs@Cellulose.^[49]^ Copyright 2021, Elsevier B.V.

He et al.^[84]^ prepared carbon nitride quantum dots (CNQDs) through a low‐temperature solid‐phase approach, where sodium citrate served as the carbon precursor and urea as the nitrogen precursor, and subsequently embedded them into a CMC matrix. The strong H‐bonding interactions between the CMC chains and CNQDs created a highly restricted environment, effectively suppressing non‐radiative relaxation processes, preserving the RTP characteristics of CNQDs, and enhancing the overall QY (Figure 23). Under 365 nm UV irradiation, the CNQDs–CMC film exhibited bright fluorescence. When the UV off, the film showed a visible yellow RTP emission to the naked eye, which gradually shifted to a green afterglow over time. Moreover, both the PL and RTP emissions of the CNQDs‐CMC film were dependent on the excitation wavelength.

**FIGURE 23:**
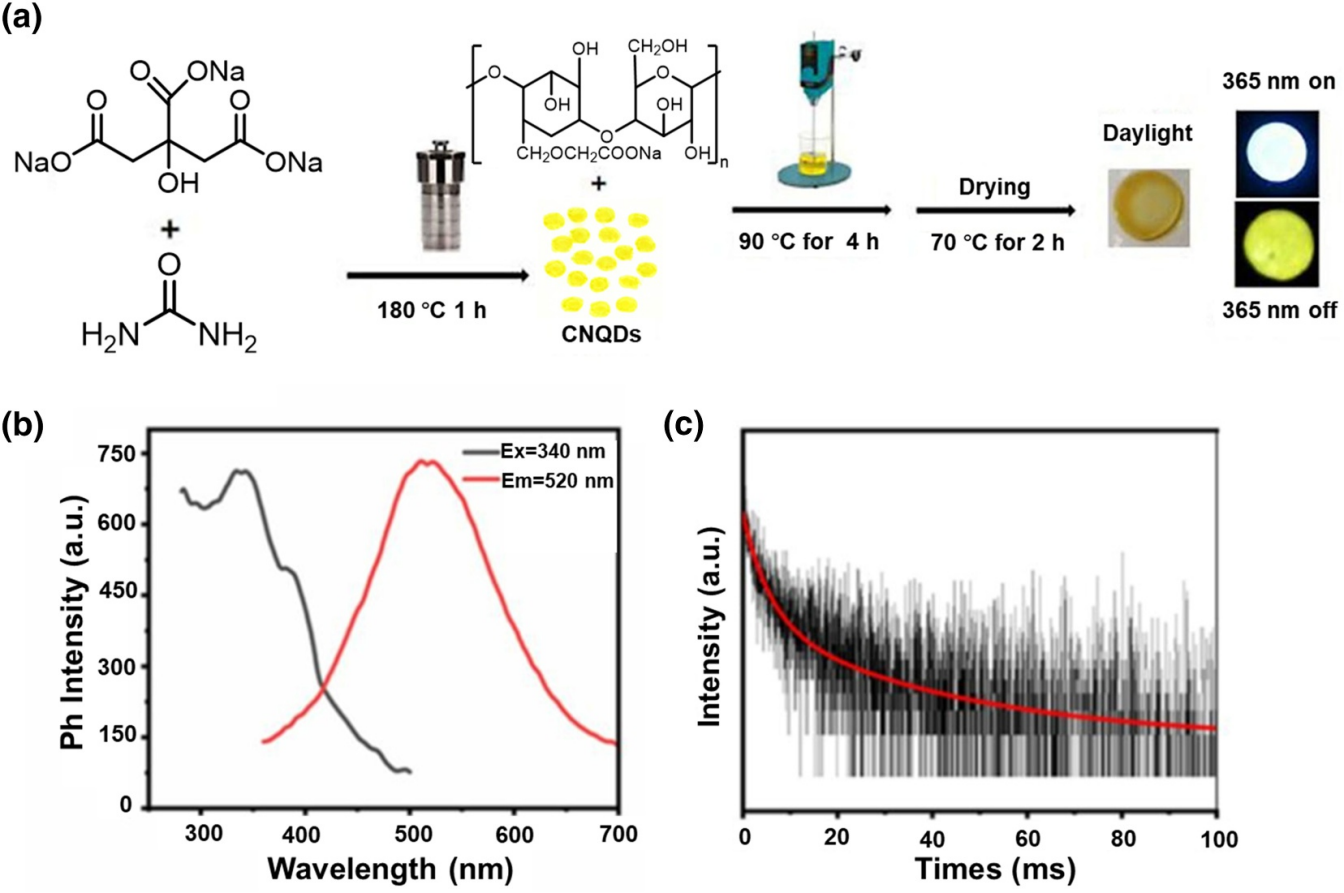
(a) Preparation of the CNQDs‐CMC film. (b) CNQDs‐CMC film RTP excitation and emission spectra. (c) RTP lifetime of the CNQDs‐CMC film.^[84]^ Copyright 2022, Elsevier B.V. RTP, room temperature phosphorescent.

Studies have shown that introducing heavy atoms into CDs can effectively enhance their ISC efficiency. Therefore, Xu et al.^[85]^ incorporated fluorinated carbon dots (FCDs) into a sodium carboxymethyl cellulose (CMCNa) matrix to obtain FCDs‐CMCNa composites (Figure 24). On one hand, owing to the high electronegativity of fluorine atoms, the doped F atoms serve as favorable binding sites for ionic interactions with the polymer matrix. These electrostatic and H‐bond interactions prevent aggregation‐induced quenching of FCDs, effectively protect triplet excitons, and suppress non‐radiative transitions. In addition, the ionic bonds are more stable than H‐bonds, which may help prevent the loss of F atoms. The resulting FCDs‐CMCNa composites exhibit good water solubility and stable yellow fluorescence, along with RTP that can be excited by both UV and visible light. Interestingly, the FCDs‐CMCNa composites display a unique temperature‐dependent optical behavior: as the temperature decreases from 300 to 90 K, both fluorescence and phosphorescence intensities reach their maximum at around 150 K.

**FIGURE 24:**
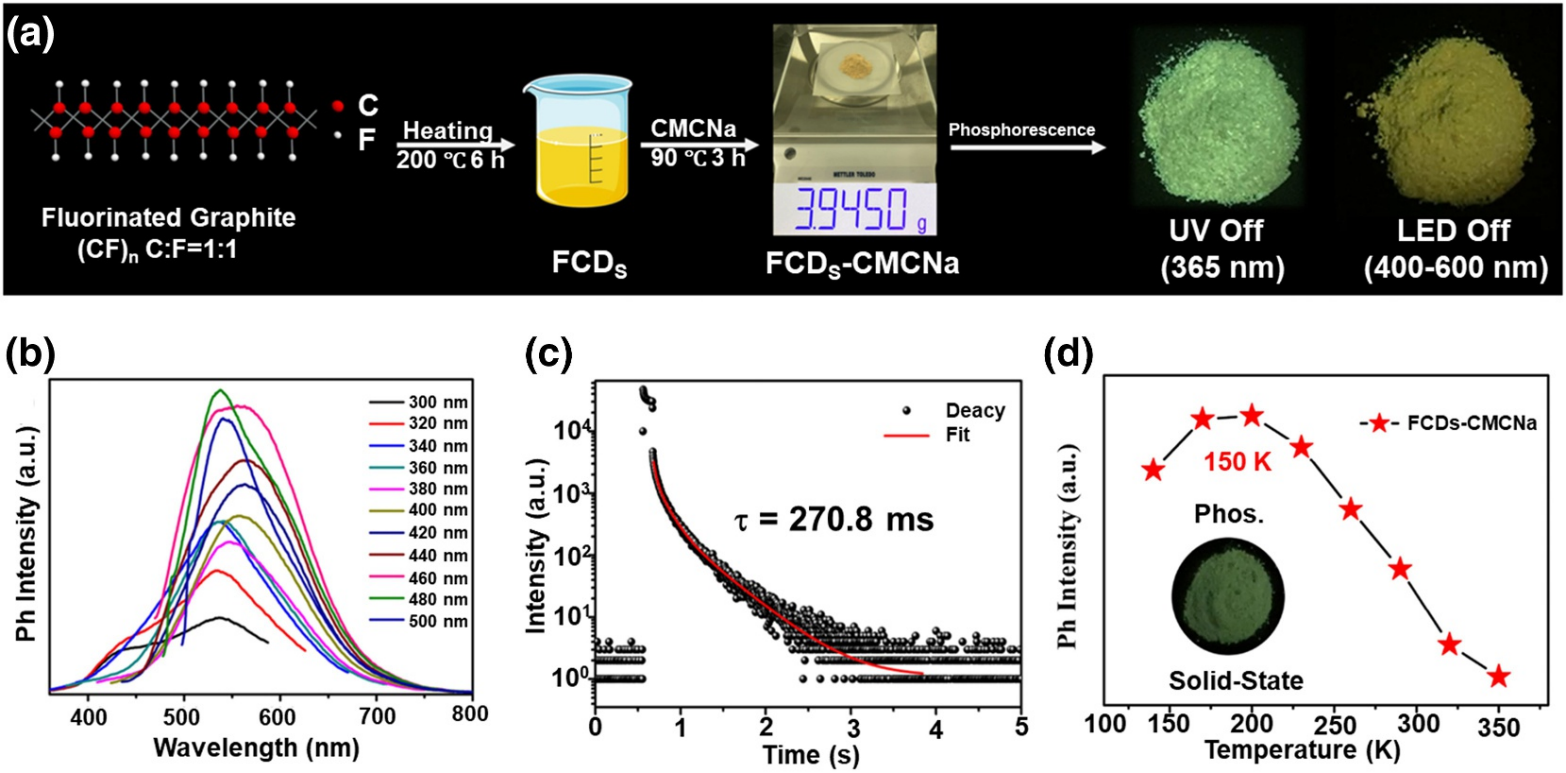
(a) Preparation for gram‐scale FCDs‐CMCNa. (b) RTP spectra of FCDs‐CMCNa. (c) RTP lifetime of the FCDs‐CMCNa. (d) Temperature dependency of RTP intensity of FCDs‐CMCNa.^[85]^ Copyright 2022, Elsevier B.V. RTP, room temperature phosphorescent.

**Supramolecular assemblies‐based guests**: Supramolecular assemblies, through the core mechanism of noncovalent interactions, endow phosphorescent materials with controllable structures, tunable properties, and enhanced stability. Zhang et al.^[86]^ employed a host‐guest interaction strategy based on macrocyclic compounds, constructing a supramolecular assembly from cucurbit[8]uril (Q[8]) and a bromophenylpyridine derivative (BPCOOH) (Figure 25). The resulting assembly was then doped into a CMC matrix to fabricate solid films. Strengthening the noncovalent interactions significantly enhanced and optimized the RTP performance of the assembly, enabling the visualization and quantitative evaluation of high‐intensity RTP in the solid state.

**FIGURE 25:**
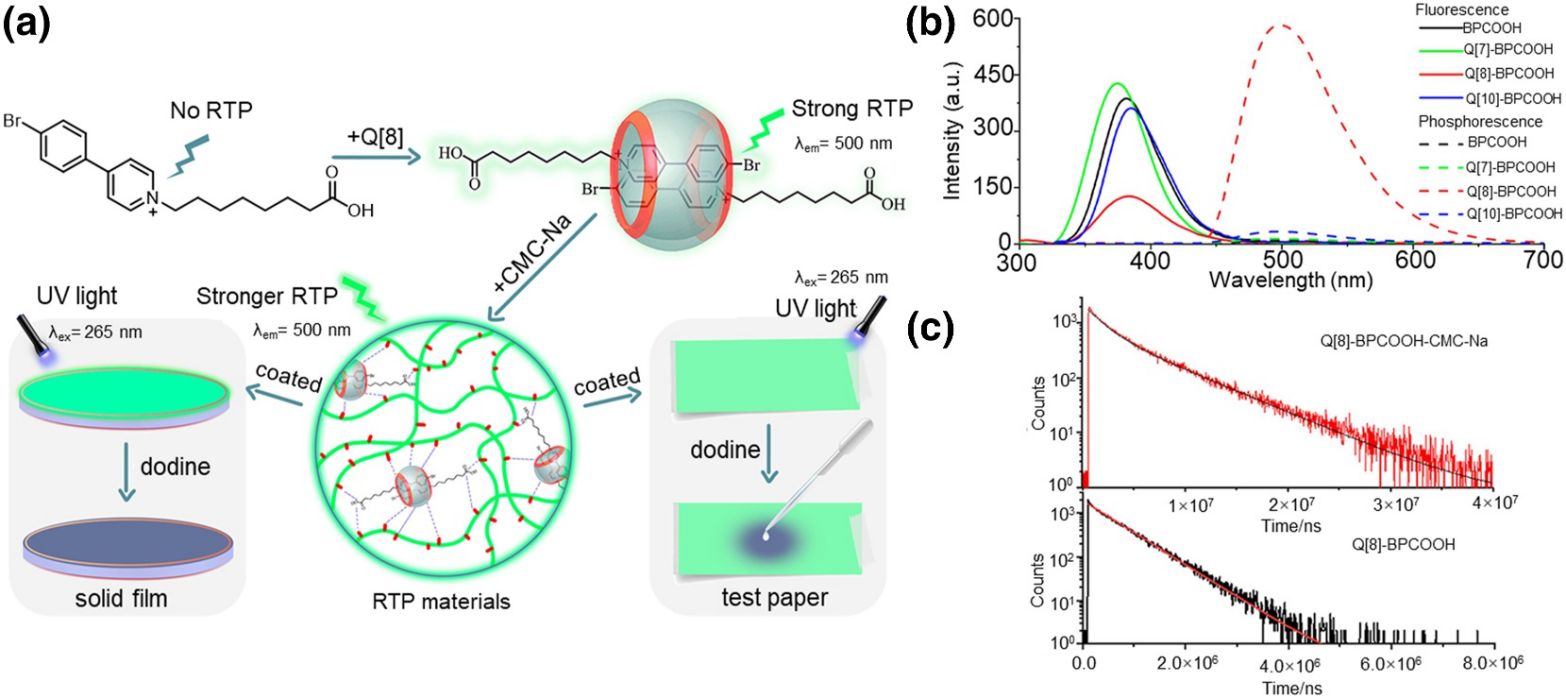
(a) Q[n]s/BPCOOH/Dodine structures and assembly structure‐application. (b) PL and RTP spectra of BPCOOH (20 μM, pH = 4.75, *λ*_ex_ = 303 nm) in 5 equiv of Q[7], Q[8], and Q[10]. (c) RTP lifetimes of Q[8]‐BPCOOH‐CMC‐Na (red) and Q[8]‐BPCOOH (black).^[86]^ Copyright 2022, American chemical society. RTP, room temperature phosphorescent.

To compare the advantages and limitations of different construction strategies, Table 1 systematically summarizes the key performance parameters of the cellulose‐based RTP materials prepared in this work. For the strategy of regulating the aggregated structure of cellulose, the material preparation is simple and feasible, enabling effective modulation of RTP properties, with emission mostly concentrated in the blue‐green region.[^23^, ^70^, ^79^] RTP materials fabricated via the strategy of reconstructing cluster emission centers involve relatively complicated preparation, but their emission spectra cover blue, green, and red colors, and ultralong phosphorescence lifetimes can be achieved in some systems; for instance, the phosphorescence lifetime of Phe9‐B reaches 2670 ms.^[72]^ Through functional modification of cellulose hydroxyl groups, diverse RTP materials with versatile structures can be designed and constructed, whose emission also covers blue, green, and red regions with excellent luminescence performance. For example, the quantum yield of CAA‐BA is as high as 45.06%,^[21]^ and the phosphorescence lifetime of sample C3 is 571.1 ms.^[76]^ For host‐guest doped cellulose‐based RTP materials, the preparation is flexible, and RTP properties can be efficiently tuned by selecting various guest molecules to realize multicolor emission. Some materials exhibit high quantum yields, such as CCYPBA@CNC with a value of 34.00%.^[80]^ Nevertheless, the four strategies each have their limitations, including narrow RTP tuning range, tedious material preparation, and complicated mechanisms. Combining multiple construction strategies can maximize the strengths of individual approaches while compensating for their shortcomings, thereby achieving precise regulation of cellulose‐based RTP materials.

**TABLE 1:** Summary of typical cellulose‐based RTP materials.

Construction strategy	Matrix	Materials	λ_ex_/λ_em_ (nm)	Color	Lifetime (ms)	PLQY (%)	Refs
3.1 Aggregation structure	MCC	MCC‐0	272/510	Green	145.3 (*τ*_3_)	3.34	[70]
		MCC‐10	272/500	Green	140.5 (*τ*_3_)	1.13	
		MCC‐12.5	272/505	Green	116.2 (*τ*_3_)	0.99	
		MCC‐15	272/505	Green	137.6 (*τ*_3_)	0.46	
		MCC‐17.5	272/505	Green	138.4 (*τ*_3_)	0.57	
	CNC	CNC‐powder	254/485	Blue	289.46 (*τ*_3_)	5.95	[67]
		CNC‐tablet	270/500	Green	322.58 (*τ*_3_)	8.27	
		CNC‐film	280/490	Green	198.26 (*τ*_3_)	3.12	
		CNC foam	350/500	Green	178.77	10.94	[71]
		CNC film	350/525	Green	344.80	5.84	
		CNC/PVA film	350/515	Green	569.38	5.15	
		Pulp film	/	/	605.43	/	
		CGAC‐pulp film	265/477	Blue	694.68	/	
		ReAC‐pulp film	275/461	Blue	808.33	/	
		ReAC‐CNCs film	275/486	Blue	631.09	/	
3.2 Cluster emission center	MCC	MCC	280/500	Green	81 (*τ*_1_)	1.45	[68]
		DAC	280/500	Green	514 (*τ*_3_)	0.26	
		DCC	280/490	Green	459 (*τ*_3_)	1.88	
		RDAC	280/500	Green	799 (*τ*_3_)	3.42	
		DC‐BA	310/490	Green	707.1	2.22	[65]
		2Bp‐B‐R	254/470	Blue	1430	1.33	[72]
		4Bp‐B‐R	254/480	Blue	1460	3.01	
		Phe9‐B‐R	310/517	Green	2670	9.37	
		1Nap‐B‐R	310/520	Green	1160	7.08	
		1Py‐B‐R	365/610	Red	210	0.84	
3.3 Hydroxyl group	MCC	/	330/505	Green	20.41	4.4	[69]
	HEC	/	330/500	Green	0.66	3.2	
	HPC	/	330/485	Blue	0.27	3.8	
	CMC	CMC‐Na	365/509	Green	114	10.7	[73]
		CMC‐Zn	365/517	Green	281	16.52	
	MCC	CMimCl	280/490	Green	85	5.47	[23]
		COHimCl	320/485	Blue	153	5.62	
		Cell‐ImCNCl (DS_t_1.24,DS_CN_0.60)	320/520	Green	158	11.81	[74]
		CPhCOOLi (DS = 0.52)	330/502	Green	254	2.96	[75]
		CBtCOOLi (DS = 0.61)	350/509	Green	433	6.04	
	CMC	C1	410∼430/520	Green	166.5	5–8	[76]
		C2	410∼430/525	Green	362.1	5–8	
		C3	410∼430/530	Green	571.1	5–8	
		C4	410∼430/620	Red	309.8	5–8	
		CMC‐Pyr_0_‐Bzp_1_	345/483	Blue	60	/	[78]
		CMC‐Pyr_0.5_‐Bzp_1_	340/617	Red	191	8.23	
		CMC‐Pyr_1_‐Bzp_1_	254/617	Red	201	2.66	
		CMC‐Pyr_2_‐Bzp_1_	335/617	Red	204	4.44	
	MCC	M1	254/480	Blue	1340	/	[66]
		M2	310/510	Green	1420	/	
		M3	310/515	Green	650	/	
		M4	365/610	Red	190	/	
		CAA‐Bp	254/496	Green	679	/	[77]
		CAA‐Na	254/524	Green	568	/	
		CAA‐Ph	254/518	Green	1251	/	
		CAA‐Py	254/609	Red	17	/	
	Cellulose (softwood pulp)	CAA‐BA	290/443	Blue	772	45.06	[21]
		CAA‐BA‐F	290/545	Green	494	/	
		CAA‐BA‐R	290/618	Red	522	/	
	MCC	CBtCOONa(4%)/CaCO_3_	310/450	Blue	244	1.32	[79]
		CBtCOONa(100%)/CaCO_3_	370/500	Green	240	3.41	
		CTPA@CNC(1/5)	370/533	Green	654	20.86	[80]
		CCYPBA@CNC(1/5)	356/521	Green	147	34.00	
	HPMC	HPMC‐FITC@NH_2_‐CAO_5:5_	365/530	Green	/	/	[81]
3.4 Host‐guest doping	α‐cellulose	MgBr_2_@ cellulose ([metal ion]:[glucose unit] = 1:1)	310/500	Green	50 (*τ*_3_)	5 (Φ_RTP_)	[82]
	MCC	CMC CDs@Cellulose	375/560	Green	167.31	/	[49]
		CA CDs@Cellulose	365/530	Green	190	/	
		SA CDs@Cellulose	365/535	Green	165	/	
	CMC‐Na	FCDs‐CMCNa	363/530	Green	270.8	5.89	[85]
		Q[8]‐BPCOOH‐CMC‐Na	302/500	Green	1.528	22.04	[86]
		CNQDs‐CMC	340/520	Green	43	6.1	[84]
	CNC	0.5CPAMBA‐Na	312/440	Blue	143.58	/	[43]
		0.1CBCA‐Na	254/488	Blue	221.02	/	
		0.5CNA‐Na	254/526	Green	140.04	/	
		0.1CPCA‐Na	254/610	Red	108.16	/	
		NP‐CNC	275/508	Green	1180	/	[83]
		PP‐CNC	275/596	Orange	300	/	
	Filter paper	ICzS2Br@filter paper	340/500	Green	1070	7.9 (Φ_*f*_)	[44]
	BC	5,7‐ICz@BC‐100	306/499	Green	793.51	8.14 (Φ_*F*_)	[42]
		5,12‐ICz@BC‐100	356/452	Blue	760.80	13.25 (Φ_*F*_)	
		11,12‐ICz@BC‐100	328/463	Blue	858.05	18.39 (Φ_*F*_)	

## 4 APPLICATIONS

Compared with traditional RTP materials, cellulose‐based RTP materials demonstrate significant advantages. Relative to organic RTP materials, their natural molecular structure eliminates the need for complex synthesis, reducing costs while avoiding issues such as biotoxicity and environmental residue. In contrast to inorganic RTP materials, they offer both flexibility and processability, overcoming the limitations associated with rigidity, while retaining renewable and degradable characteristics. Leveraging these properties, they show promising application potential in fields such as bioimaging, information encryption, and environmental monitoring. This section will summarize the current applications of cellulose‐based RTP materials in various domains, aiming to provide guidance for their further development.

### 4.1 Information encryption

With the advancement of information security, multilevel information encryption has been widely applied in the field of anti‐counterfeiting labels.[^87^, ^88^, ^89^] Different cellulose‐based RTP materials usually exhibit distinct phosphorescence lifetimes. By integrating these materials in a rational manner, multilevel encryption and advanced anti‐counterfeiting can be achieved. For example, MCC and the reconstructed cluster emission centers DC and DC‐BA exhibit distinct phosphorescence lifetimes. These materials were patterned into “88”‐shaped modules.^[65]^ Under 365 nm UV irradiation, the number “88” displayed bright blue fluorescence. When the UV was off, the “88” pattern immediately switched to an afterglow mode. Over time, the numbers gradually changed from “88” to “96,” and eventually to “15.” This time‐dependent RTP evolution enables dynamic information encoding, offering great potential for multilevel data encryption (Figure 26a). The biocompatible and food‐safe RTP material ICz@BCs is suitable for use in anti‐counterfeiting applications for food packaging.^[42]^ Three white star‐shaped patterns made from different ICz@BCs exhibit blue emission under UV excitation, serving as the first‐level anti‐counterfeiting feature. When the excitation is removed, the persistent afterglow from the patterns provides a second‐level anti‐counterfeiting feature. As time passes, the number of glowing stars gradually decreases until no emission is observed, representing the third‐level anti‐counterfeiting stage (Figure 26b).

**FIGURE 26:**
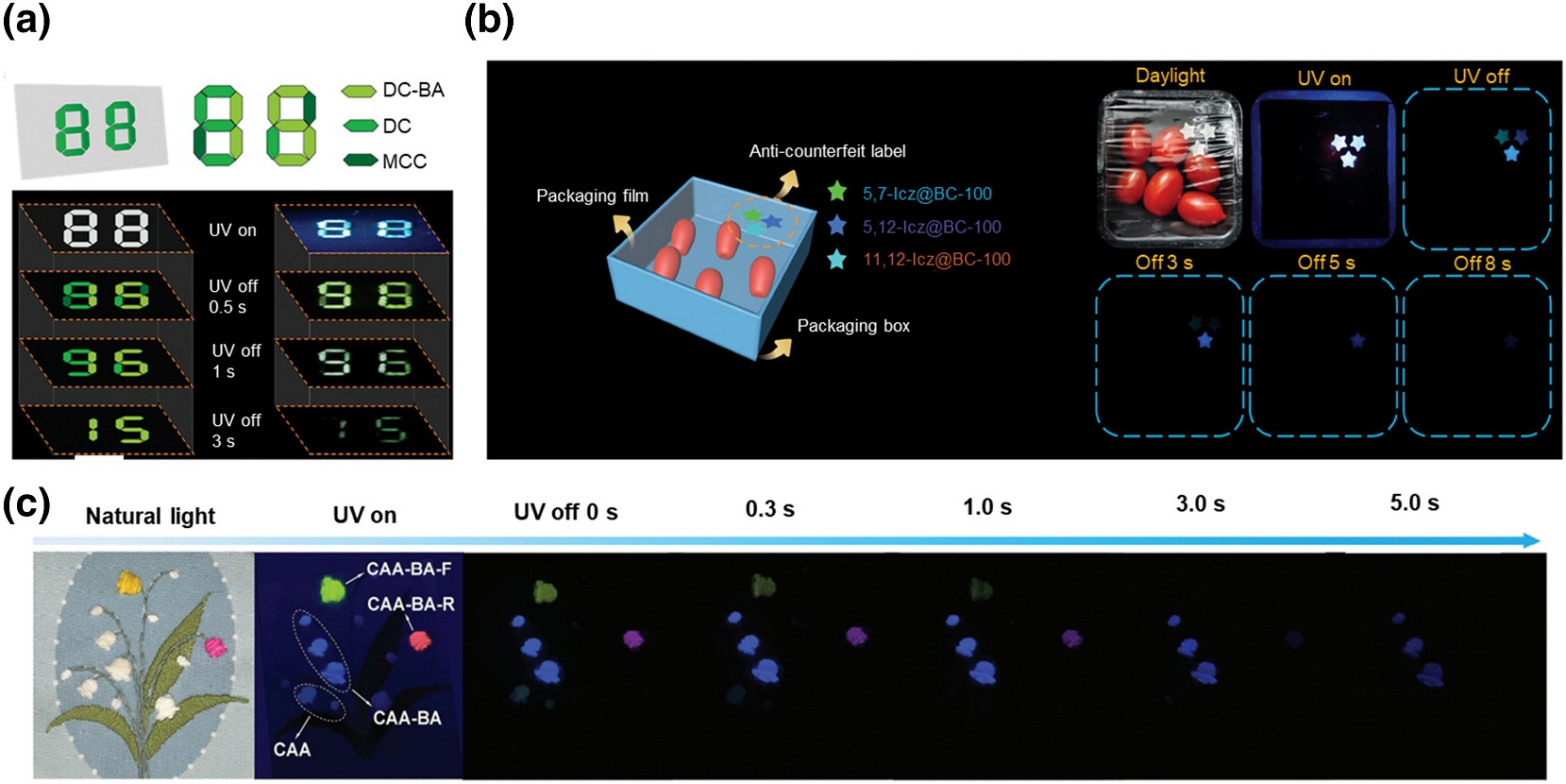
Information encryption achieved by MCC, DC, and DC‐BA.^[65]^ (b) Application of anti‐counterfeiting labels on food packaging.^[42]^ (c) Photos of embroidery patterns made with Room temperature phosphorescent filaments under 254 nm UV light and after the cessation of irradiation.^[21]^ Copyright 2024, John Wiley and Sons.

In addition to time dependence, the afterglow color of cellulose‐based RTP materials provides an additional dimension for advanced anti‐counterfeiting applications.^[80]^ The RTP color can be conveniently tuned through an efficient FRET process, thereby expanding its practical applications in anti‐counterfeiting. For example, floral patterns can be embroidered using CAA, CAA‐BA, and conventional cellulose filaments with different emission colors.^[21]^ When exposed to UV light, all filaments exhibit fluorescence. After turning off the UV light, each filament showed distinct afterglow duration and color, demonstrating great potential for use in advanced anti‐counterfeiting labels (Figure 26c).

RTP materials with humidity responsiveness and biocompatibility are of great significance for authenticity identification in food packaging and artworks.[^21^, ^42^, ^49^] Wang et al. printed QR codes on labels using cellulose doped with humidity‐responsive long‐afterglow CDs.^[49]^ Under humidity levels of 50% or lower, the long afterglow of the QR code allows information retrieval via smartphone scanning even after UV excitation is removed. However, when the humidity increases to 75% or higher, the afterglow of the QR code almost disappears after removing the excitation source, rendering the code unreadable (Figure 27a).

**FIGURE 27:**
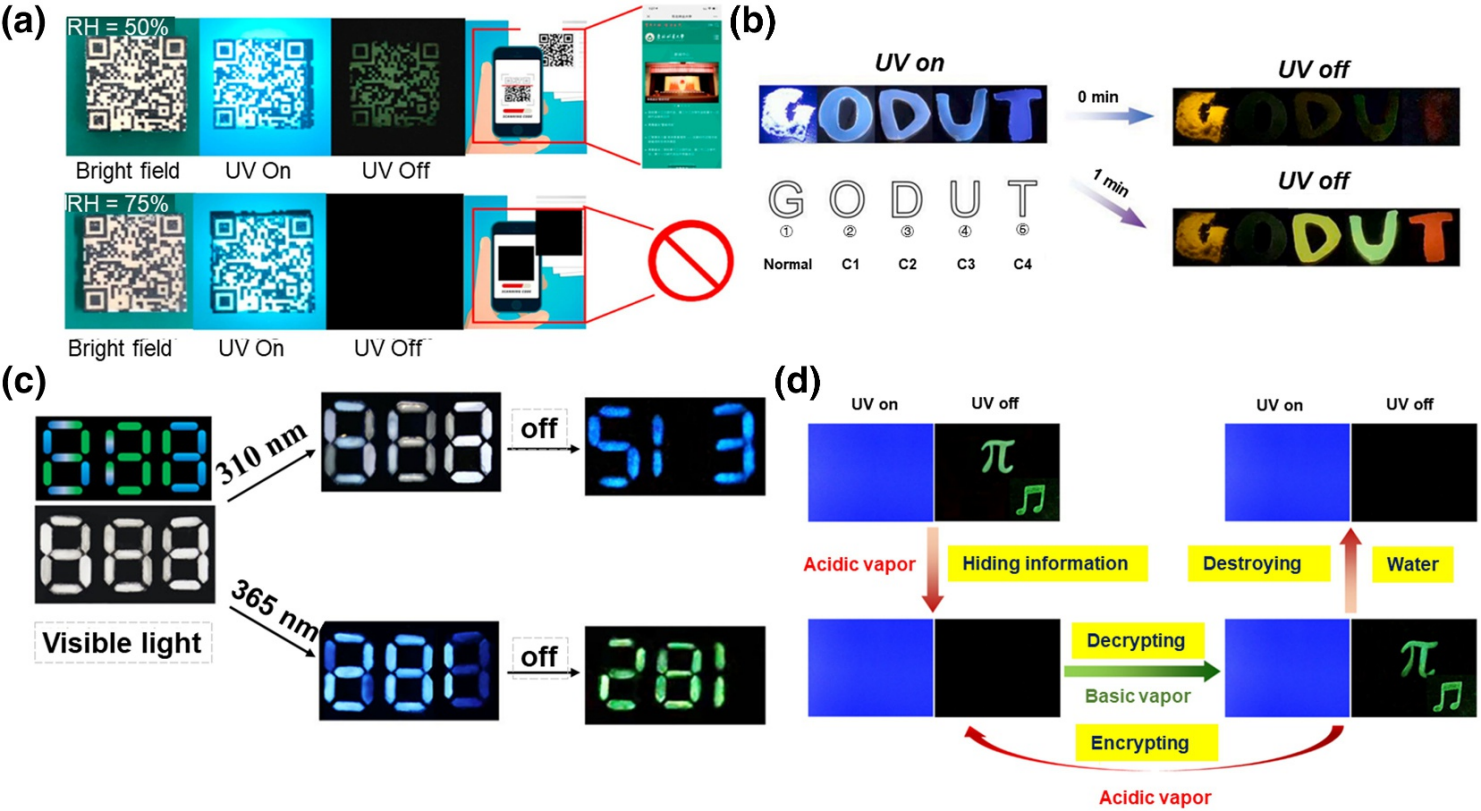
(a) Photos of QR codes printed with CMC CDs@Cellulose under various relative humidity conditions, with the far‐right image showing smartphone scanning of the afterglow.^[49]^ Copyright 2021, Elsevier B.V. (b) Multiple data encryption with normal p compound (Normal) and C1–C4.^[76]^ Copyright 2022, Elsevier B.V. (c) Anticounterfeiting code with three inks.^[79]^ Copyright 2022, Springer nature limited. (d) Cellulose‐based phosphorescent material application (hidden phosphorescent patterns, *λ*_ex_ = 365 nm).^[75]^ Copyright 2022, Chinese chemical society.

In addition, some materials with photo‐enhanced RTP properties can dynamically regulate their phosphorescence lifetime by varying the UV irradiation time. Zeng et al.^[76]^ reported that CMC grafted with bulky aromatic derivatives of different conjugation degrees (C1‐C4) exhibited photo‐enhanced p‐RTP behavior. By combining these with conventional p‐RTP emitters, multilevel data encryption could be achieved. As illustrated in Figure 27b, the “GODUT” pattern was fabricated by assembling the letter “G” using a common p‐RTP compound and “ODUT” using C1‐C4 RTP materials. Under UV on, a bright blue “GODUT” pattern was clearly visible. When the UV was suddenly switched off, only the yellow afterglow of “G” remained as a static image. Upon continuous UV for more than 1 min, the consumption of triplet oxygen significantly prolonged the phosphorescence lifetime of C2‐C4. Consequently, when the UV was off, the “GDUT” pattern could be directly observed, demonstrating multilevel data encryption and anti‐counterfeiting capability.

Excitation dependence is also a common characteristic exhibited by cellulose‐based RTP materials, enabling the encoded information in phosphorescent patterns to be controlled by varying the excitation source.[^72^, ^78^] By exploiting the aggregation‐dependent emission behavior of CBtCOONa, You et al.^[79]^ prepared cellulose‐derived inks with blue, green, and excitation‐dependent phosphorescence to print encoded patterns. As shown in Figure 27c, the printed code displayed the blue number “513” under 310 nm excitation, which transformed into the green number “281” when excited at 365 nm.

Some cellulose derivatives special exhibit pH‐responsive phosphorescence and excellent processability, allowing them to be used as water‐based inks for facile inkjet printing of multiple patterns in advanced information encryption applications. For example, anionic cellulose derivative (CBtCOOLi) inks were used to print patterns on a blue‐fluorescent paper. After switching off the UV light, distinct green phosphorescent patterns became visible (Figure 27d).^[75]^ When the printed paper was exposed to HCl vapor, the green phosphorescent patterns disappeared. Subsequently, treatment with NH_3_·H_2_O vapor restored the green phosphorescent emission. This process exhibited excellent reversibility and reproducibility. Moreover, the printed patterns could be easily destroyed by applying a few drops of water, enabling convenient erasure of confidential information. Such stimulus‐responsive on/off switching behavior provides a simple yet effective strategy for information encryption and protection.

To clearly identify the phosphorescent information, RTP materials used for anti‐counterfeiting and encryption are typically observed under dark conditions. Therefore, developing systems that can achieve efficient anti‐counterfeiting performance under both daylight and dark environments has become an emerging research focus. By exploiting the left‐handed chiral nematic structure of CNC films and co‐assembling polyethylene pyrrolidone (PVP) with aromatic sodium salts, Zhou et al. fabricated composite films that exhibit vivid structural colors visible under daylight conditions.^[43]^ As shown in Figure 28a, under sunlight, the number “88” displays angle‐dependent structural colors that vary with the viewing direction. Under irradiation with a 254 nm UV, a distinct “88” pattern composed of blue, violet, and cyan colors became visible. After the UV light was turned off, the “88” pattern gradually changed in color from blue, green, yellow, to cyan; after 2.5 s, it evolved into the number “33”, and only a clear “3” remained after 6 s. This result demonstrates that the film possesses dual anti‐counterfeiting capability under both daylight and dark conditions. Wang et al.^[83]^ took advantage of the circular dichroism of CNC films to construct composite CNC‐based films exhibiting pronounced structural color, RTP, and CP‐RTP, enabling their application in multichannel information encryption. As illustrated in Figure 28b, an information panel composed of a 3 × 3 pixel array was fabricated using four types of CNC films (PP‐CNC‐396, NP‐CNC‐640, NP‐CNC‐409, and CNC‐400). Owing to the distinct structural colors of the films, the array clearly displays the letter “H” under sunlight. When the UV light was turned off, three phosphorescent films (PP‐CNC‐396, NP‐CNC‐640, and NP‐CNC‐409) exhibited RTP emission that reveals the letter “σ”. The PP‐CNC‐396 film containing pyrene emits red RTP that fades rapidly due to its short lifetime, causing the array to evolve into the letter “T” after 3 s. Because NP‐CNC‐640 and NP‐CNC‐409 display CP‐RTP with opposite chiralities, the letter “V” and the punctuation mark “:” can be selectively observed under right‐ and left‐handed circular polarization filters, respectively. Therefore, this 3 × 3 pixel array enables five independent channels of data storage and encryption.

**FIGURE 28:**
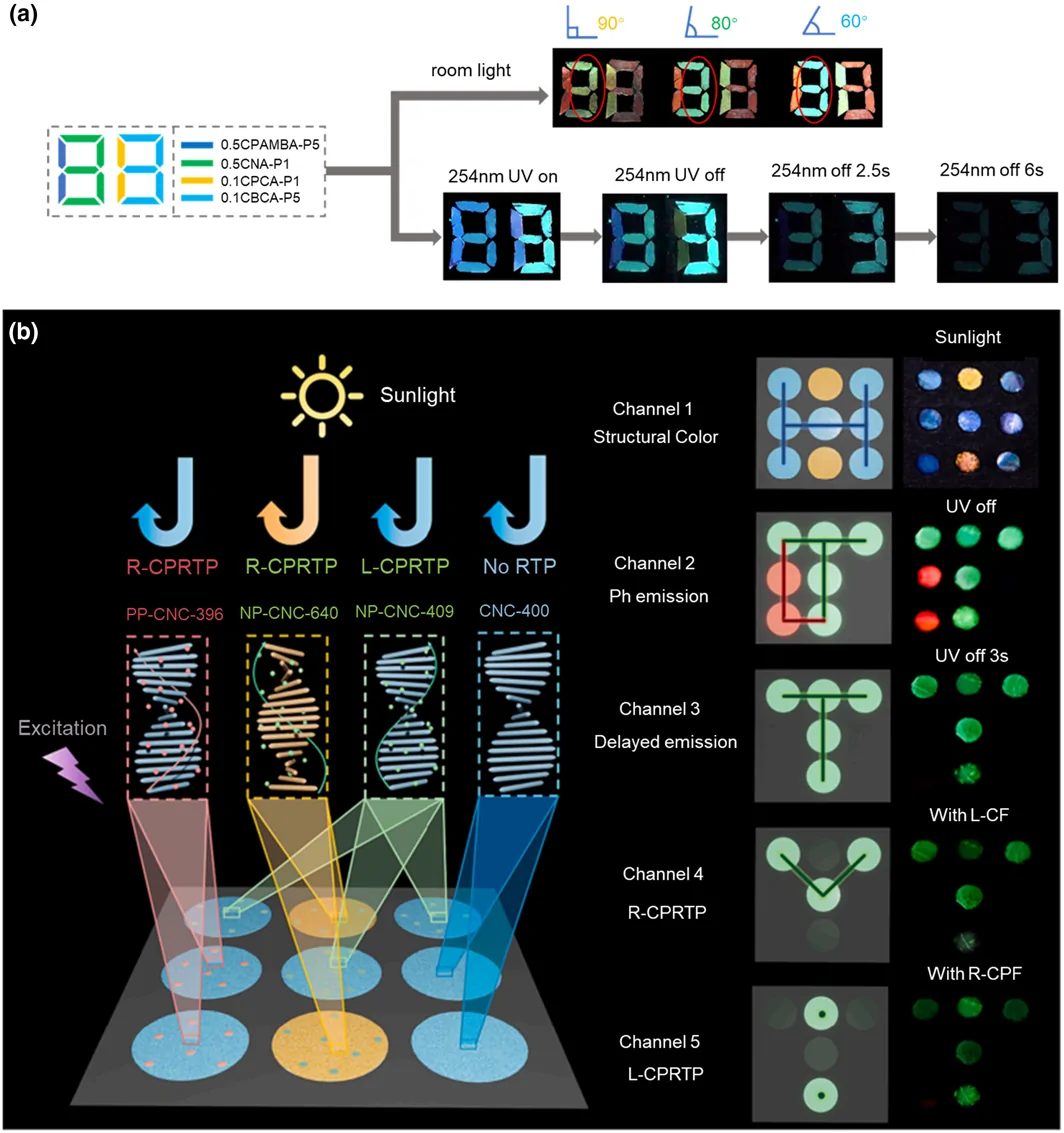
(a) Anticounterfeiting performance under sunlight and darkness for various encrypted data.^[43]^ Copyright 2024, American chemical society. (b) Multilevel information encryption realized by a 3 × 3 pixel array on a hybrid CNC film, utilizing combined structural color and afterglow signals.^[83]^ Copyright 2024, John Wiley and Sons.

### 4.2 Bioimaging

The chemical composition of plant cell walls has been analyzed using imaging techniques such as fluorescence labeling.[^90^, ^91^] As cellulose is the primary component of plant cell walls and is widely distributed in plant tissues, its persistent phosphorescent properties can be utilized for label‐free tissue imaging. By drying sections of various plant tissues and observing them with a smartphone‐based imaging device, persistent emission was detected in different tissues such as cucumber, carrot, lotus root sprouts, and Chinese cabbage.^[45]^ Compared with imaging under UV excitation, time‐resolved imaging after turning off the UV light provided a darker background, effectively eliminating autofluorescence and scattering without requiring complex time‐gating equipment. The RTP lifetime and color varied among different tissue sections, which may be related to differences in cell types, density, and endogenous components. This label‐free imaging approach holds promise for broader applications in plant tissue analysis, and the phosphorescent materials derived from edible plants are safer for both humans and the environment (Figure 29a).

**FIGURE 29:**
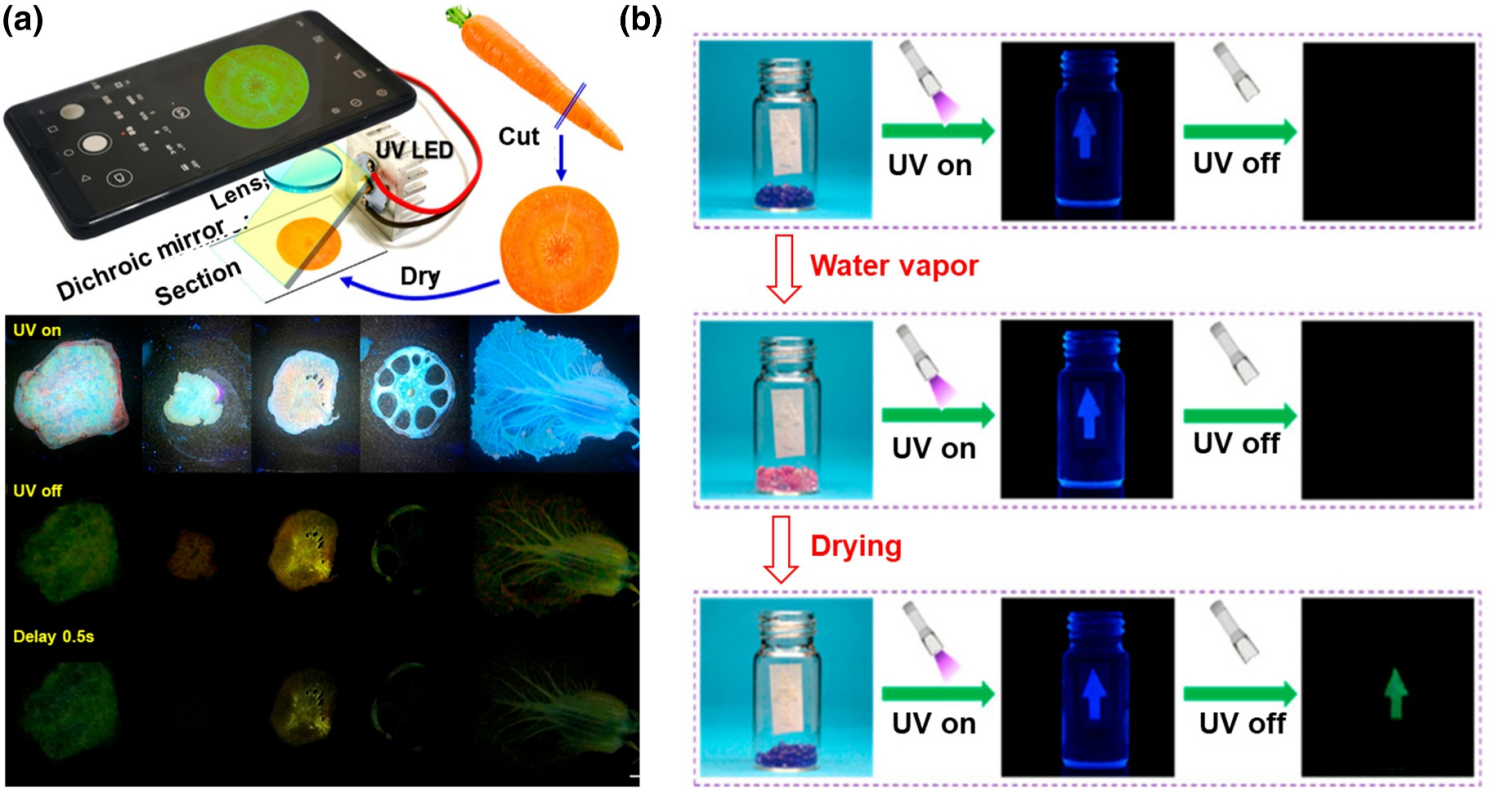
(a) Label‐free luminescence imaging of plant tissues on a smartphone‐based apparatus, demonstrating dried specimens (from left to right: cucumber, turnip, carrot, lotus sprout, and Chinese cabbage) extracted from Videos 3−7. Scale bar = 2 mm.^[45]^ Copyright 2024, American chemical society. (b) Application of a COHimCl‐based phosphorescent label in environment monitoring under 365 nm UV light.^[23]^ Copyright 2022, American chemical society.

### 4.3 Environmental monitoring

For humidity‐sensitive materials, foods, and pharmaceuticals, variations in ambient humidity are critical to their storage stability and practical performance.[^21^, ^77^, ^92^] A cellulose derivative, 1‐(2‐hydroxyethyl)imidazolium chloride cellulose (COHimCl), synthesized via a two‐step homogeneous derivatization process, exhibits irreversible humidity‐responsive RTP behavior, enabling visual monitoring of excessive humidity exposure.^[23]^ As illustrated in Figure 29b, a COHimCl tag obtained by acetone precipitation and a color‐changing silica gel indicator were placed together in a sealed vessel. Initially, the COHimCl tag showed no phosphorescence, while the silica gel appeared blue. Upon exposure to water vapor, the silica gel turned red, and the COHimCl tag remained non‐emissive. After drying to remove residual moisture, the silica gel reverted to blue, and the COHimCl tag exhibited bright green phosphorescence. Because this humidity‐induced phosphorescence process is irreversible, the appearance of emission indicates that the storage environment has previously experienced a high‐humidity condition. Such a visible, non‐falsifiable phosphorescent tag ensures authenticity and provides a unique visual record of humidity history.

## 5 SUMMARY AND OUTLOOK

This review provides a comprehensive summary of recent advances in constructing RTP materials based on cellulose and its derivatives, covering aspects from mechanistic understanding to strategy optimization and application exploration. At the mechanistic level, it is clarified that the RTP emission of cellulose—an atypical phosphorescent material—originates from clusteroluminescence. In this process, the overlap between π or lone‐pair electrons within the cluster extends the conjugation of electron clouds and enhances molecular rigidity, leading to efficient RTP emission. This mechanism also provides valuable insight for studying other nonconventional biomass‐based RTP materials. From the perspective of material design, the current research progress can be summarized into four major strategies: adjusting the aggregation state of cellulose, reconstructing cluster emission centers, functionalizing hydroxyl groups, and constructing host‐guest doping systems. These approaches provide systematic pathways to expand the performance boundaries of RTP materials. Each of these strategies offers distinct advantages and can be synergistically combined, jointly advancing the development of cellulose‐based RTP materials. In terms of applications, cellulose‐based RTP materials, featuring low cost, excellent biocompatibility, and tunable structural properties, have shown great potential in information encryption, bioimaging, and environmental monitoring.

Overall, the research challenges of cellulose‐based RTP materials can be classified into several aspects. First, more advanced equipment is needed to deeply explore the emission mechanism under the complex molecular structure based on the existing CTE mechanism. Secondly, due to the lack of conjugated systems in cellulose molecules, its RTP mainly originates from oxygen‐cluster emission centers formed by the clustering effect along molecular chains.^[50]^ The relatively high T_1_‐S_0_ energy gap of such cluster chromophores restricts RTP to the blue and green regions, making it difficult to achieve red or near‐infrared phosphorescence.^[69]^ However, strategies including reconstructing cluster emission centers in cellulose, modifying cellulose with conjugated moieties, and doping cellulose with guest molecular modules featuring π‐conjugation, low triplet energy levels, and strong SOC effects can reduce the energy gap of clustering centers, enhance SOC effects, and rigidify the molecular microenvironment.[^78^, ^81^, ^85^] In this way, near‐infrared RTP can be realized, endowing such cellulose‐based materials with promising application prospects in bioimaging,[^93^, ^94^] photodynamic therapy,^[95]^ monitoring and sensing,^[23]^ and other fields. Third, the development of materials with both ultra‐long phosphorescence lifetimes and high QY is insufficient, and further exploration of regulation strategies is required. It is anticipated that this review will provide valuable guidance and strategies for developing cellulose‐based RTP materials with multicolor tunability, prolonged lifetimes, and high QY.

## CONFLICT OF INTEREST STATEMENT

The authors declare no conflicts of interest.
